# Nuclear Magnetic Resonance Spectroscopic Analysis of the Evolution of Peroxidation Products Arising from Culinary Oils Exposed to Thermal Oxidation: An Investigation Employing ^1^H and ^1^H-^1^H COSY and TOCSY Techniques

**DOI:** 10.3390/foods11131864

**Published:** 2022-06-24

**Authors:** Gilbert Ampem, Adam Le Gresley, Martin Grootveld, Declan P. Naughton

**Affiliations:** 1 Department of Chemistry and Pharmaceutical Sciences, SEC Faculty, Kingston University, Kingston-upon-Thames, Surrey KT1 2EE, UK; g.ampem@kingston.ac.uk (G.A.); d.naughton@kingston.ac.uk (D.P.N.); 2 Leicester School of Pharmacy, De Montfort University, Leicester LE1 9BH, UK; mgrootveld@dmu.ac.uk

**Keywords:** aldehydes, culinary oil, continuous thermo-oxidation, discontinuous thermo-oxidation, lipid oxidation products (LOPs), proton nuclear magnetic resonance (^1^H NMR) analysis

## Abstract

Scientific warnings on the deleterious health effects exerted by dietary lipid oxidation products (LOPs) present in thermally stressed culinary oils have, to date, not received adequate attention given that there has been an increase in the use and consumption of such oil products in everyday life. In this study, high-resolution ^1^H nuclear magnetic resonance (NMR) analysis was used to characterize and map chemical modifications to fatty acid (FA) acyl groups and the evolution of LOPs in saturated fatty acid (SFA)-rich ghee, monounsaturated fatty acid (MUFA)-rich groundnut, extra virgin olive, and macadamia oils, along with polyunsaturated fatty acid (PUFA)-rich sesame, corn and walnut oils, which were all thermally stressed at 180 °C, continuously and discontinuously for 300 and 480 min, respectively. Results acquired revealed that PUFA-rich culinary oils were more susceptible to thermo-oxidative stress than the others tested, as expected. However, ghee and macadamia oil both generated only low levels of toxic LOPs, and these results demonstrated a striking similarity. Furthermore, at the 120 min thermo-oxidation time-point, the discontinuous thermo-oxidation episodes produced higher concentrations of aldehydic LOPs than those produced during continuous thermo-oxidation sessions for the same duration. On completion of the thermo-oxidation period, a higher level of triacylglycerol chain degradation, and hence, higher concentrations of aldehydes, were registered in culinary oils thermally stressed continuously over those found in discontinuous thermo-oxidized oils. These findings may be crucial in setting targets and developing scientific methods for the suppression of LOPs in thermo-oxidized oils.

## 1. Introduction

### 1.1. Background

Culinary oils are prone to peroxidative degradation cycles under varying conditions. The oxidation of culinary oils is strongly promoted by heat, which is by far the most common and significant facilitator in view of their vast use for frying/cooking episodes at both industrial and domestic levels. In addition to heat, some metal ions, photosensitizing pigments, and most especially free radicals and light all represent factors that accelerate the peroxidation of unsaturated fatty acids (UFAs) in culinary oils. This peroxidation process causes oil deterioration, which impacts negatively on their nutritional value and organoleptic properties, reduces their shelf-lives and, most importantly, promotes the evolution of deleterious oxidation products therein. These harmful oxidation products, also referred to as lipid oxidation products (LOPs), have been reported to act as promoters or perpetuators of a wide range of human diseases, and these include, but are not limited to, cardiovascular conditions, cancer, congenital malformations, gastropathic diseases, hypersensitivity, and neurodegenerative and inflammatory disorders [1]. Moreover, they also exert significant teratogenic effects [1].

Despite many scientific warnings available on LOPs generated in oxidized, UFA-rich culinary oils during frying or cooking processes, the perceived and acclaimed health benefits offered by these frying media have led to a surge in their consumption [2,3,4]. For example, the global consumption of palm, soybean, sunflower, palm kernel, groundnut, cottonseed, coconut, olive and rapeseed oils has increased from 166.76 to 207.93 million metric tons from 2013/2014 to 2020/2021 [4]. A large percentage of the human population is exposed to LOPs via their dietary consumption and/or the inhalation of fumes during frying/cooking episodes. This is ascribable to their processing at high temperatures, typically at cooking temperatures of approximately 180 °C.

Thermo-oxidation of UFA-rich culinary oils at 180 °C has been reported to generate several types of LOPs at high millimolar concentrations [5,6]. Indeed, the nature and type, as well as concentrations of LOPs produced, are dependent on the saturation degree of the culinary oils employed. As expected, the evolution of LOPs in culinary oils is in order of polyunsaturated fatty acids (PUFAs) > monounsaturated fatty acids (MUFAs) > saturated fatty acids (SFAs), observations which have been confirmed by many previous studies [2,5,6,7]. In addition, the thermo-oxidation frying temperature, its duration and the type of frying episode employed represent important factors that influence the dynamics of chemical changes and the rate of evolution of LOPs in peroxidizing culinary oils [6,8,9].

Several scientific studies have employed many standard methods for the identification and characterization of LOPs in culinary oils. These standard methods include peroxide value, conjugated dienes and trienes, *para*-anisidine value, thiobarbituric acid-reactive substances, and free fatty acids, iodine value, along with tocopherol and total phenolic contents [6,10,11]. However, these alternative methods lack specificity, which renders it difficult to identify individual compounds. This often gives rise to inaccurate interpretations of data and unreliable conclusions. Moreover, the applications of data obtained from the use of these methods may also be inefficient. Additionally, these methods are laborious and onerous and therefore require considerable time and effort to set up and run [6,11,12]. The thiobarbituric-acid-reactive substances assay also artefactually generates a variety of primary and secondary LOPs in thermally stressed culinary oils [1]. In contrast, one- and two-dimensional nuclear magnetic resonance (NMR) spectroscopies are established techniques that are efficient and specific for the identification of individual LOPs in oils [1,12]. Furthermore, the practical importance of NMR spectroscopy is further exemplified by applications of Pure Shift Yielded by Chirp Excitation (PSYCHE) and hyphenated diffusion techniques (PSYCHEiDOSY) for the resolution of small molecule resonances in complex multicomponent mixtures [13].

### 1.2. Rationale

The present study is a further contribution to the report by [6], which was the first scientific report on coconut oil, olive oil, rapeseed oil and sunflower oil exposed to thermal stressing episodes both continuously and discontinuously at 180 °C. According to this report, sunflower oil, which was highly susceptible to continuous thermo-oxidation, was also the most resistant product amongst the three PUFA-rich oils analyzed against discontinuous thermo-oxidation episodes. The degree of resistance to discontinuous thermo-induced autocatalytic oxidation cycles by sunflower oil was explained to be innate rather than the expectation of this being linked to the degree of unsaturation of the oil [6]. The authors also concluded that discontinuous thermo-oxidation episodes, which were initially expected to generate smaller amounts of LOPs in comparison to those observed during continuous thermo-oxidation processes, rather led to the evolution of higher levels of LOPs in the studied culinary oils [6].

As a continuation of these studies, ghee, as well as groundnut, extra virgin olive, macadamia, sesame, corn and walnut oils, were investigated in accordance with the scientific protocol described in [6]. In addition, the research questions outlined in the groundwork of this report were addressed in the current study by the use of culinary oils that were not investigated by [6]. This study also mapped the corresponding chemical modifications of acyl groups of continuous and discontinuous thermo-oxidized culinary oils. Furthermore, the evolution of LOPs in all studied oils was monitored as a function of both continuous and discontinuous thermo-oxidation processes at a frying temperature of 180 °C. Chemical modifications observed in the fatty acid acyl chains, and the degree of evolution of LOPs in thermally stressed culinary oils, were used to profile their thermo-oxidative susceptibilities. In addition, two-dimensional ^1^H-^1^H correlation and total correlation spectroscopies (COSY and TOCSY, respectively) were also employed to aid the identification of LOPs present in the thermo-oxidized oils.

## 2. Materials and Methods

### 2.1. Culinary Oil Samples

Culinary oils of varying unsaturation degrees (Table 1) were procured from a local supermarket in London, United Kingdom. Prior to analysis, all oil samples were retained under dark room storage, and under the same ambient temperature conditions, for not more than a 72 h duration. Table 1 shows the percentage compositions of the key FAs of all procured culinary oil samples obtained from their respective product labels; these are compared with those determined by ^1^H NMR analysis, along with their corresponding iodine values computed using this technique. Also stipulated on the culinary oil product labels was the claim that ghee constitutes 99.8% milk fat and therefore represents a ‘pure butter ghee’. Corn oil was described as a ‘100% pure oil’, and macadamia oil as a ‘cold-pressed, extra virgin oil’ containing 6.2 mg vitamin E in a 250 mL bottle, which constitutes 51% of the 12 mg vitamin E threshold, as stipulated in the legal daily Nutrient Reference Value (NRV) for this nutrient.

According to the ^1^H NMR analysis of the FA group compositions and iodine values (IVs), ghee was indeed classified as an SFA-rich oil. Groundnut, extra virgin olive and macadamia oils, however, were classified as MUFA-rich oils. Interestingly, sesame, corn and walnut oils were all labeled as PUFA-rich oils. However, the high content of ω-3 FAs and Ln FAs, as well as PUFAs, which are predominantly present as triacylglycerols in walnut oil, could also render the oil to be more susceptible to peroxidation than corn and sesame oils. Hence, this could give rise to a differential pattern of secondary aldehydic LOPs in thermally stressed walnut oil than in those arising from the latter oil source [6].

### 2.2. Thermal Stressing of Culinary Oil Samples

Thermal stressing of ghee, groundnut, extra virgin olive, macadamia, sesame, corn and walnut oils was conducted in the presence of atmospheric O_2_ at 180 °C using standard heating or cooking practices. The same types of 100 mL beakers, electronic balance (±0.1 g accuracy, Mettler (United Kingdom, UK), Model AT261) and electronically controlled hot-plate (230 V, 50 Hz, 750 W; Stuart heat-stir (UK), Model SB162) were used for the weighing and heating experiments, respectively, for comparative quantitative ^1^H NMR evaluations (as described by [5,6]. All glassware was dried under nitrogen (N_2_) prior to its use in thermo-oxidation processes. Culinary oil bottles were completely sealed after discharging the amount of oil required for thermo-oxidation experiments [6]. 

### 2.3. Continuous Thermo-Oxidation Method

The continuous thermo-oxidation method initially described by [6] was adopted and modified somewhat. A 20.0 g amount of accurately weighed culinary oils were transferred into a 100 mL beaker with surface area of 149.23 cm^2^. The culinary oil, with a surface area in the beaker of 39.27 cm^2^, was thermally stressed at 180 °C on an electronically controlled hot plate for a 300 min duration. Subsequently, 0.30 mL aliquots of heated culinary oils were directly sampled into clear, easy open, Cole-Parmer microcentrifuge reaction vials (2.0 mL volume, polypropylene). Oil samplings were performed at regular 30 min intervals using a graduated 230 mm Fischerbrand glass Pasteur pipette. Each sampled oil was immediately cooled on ice and then processed for ^1^H NMR measurements. Corresponding unheated culinary oils served as controls for all thermo-oxidized oils. All sampling withdrawals of these oil samples were not replenished. Continuous thermo-oxidation episodes were repeated as three separate replicates for each culinary oil product tested.

### 2.4. Discontinuous Thermo-Oxidation Method

The discontinuous thermo-oxidation protocol originally described by [6] was also adopted for the experiments described here, with a small modification. Briefly, 20.0 g of each oil was thermally stressed at 180 °C in 100 mL beakers for a 30 min period and then allowed to cool to ambient temperature, at which it was maintained for a period of 120 min, prior to being exposed to thermal stressing episodes again at 180 °C for 30 min. This procedure was repeated three times for each oil investigated so that there were *n* = 3 replicates per oil type. Thermo-oxidation of the oils was conducted at 180 °C on an electronically controlled hot plate. Aliquots of 0.30 mL of each culinary oil were sampled at 30, 180, 330 and 480 min time-points. Oils sampled at these time-points were rapidly cooled on ice. When cooled to ambient temperature, oils were sampled at time-points of 90, 120, 210, 300, 390, and 450 min into polypropylene vials post-heating. As noted above, withdrawn oil samples were not replenished during the discontinuous thermo-oxidation episodes. Unheated culinary oils served as control experiments for their corresponding thermo-oxidized samples. 

### 2.5. ^1^H NMR Measurements

Changes in the composition of FAs and the evolutions of LOPs in sampled thermally stressed culinary oils were monitored by ^1^H NMR analysis conducted on Bruker Avance III 600 MHz (TXI) spectrometer (Kingston University, London, UK) operating at a ^1^H frequency of 600.13 MHz. The pre-set acquisition parameters of this 14.1 T spectrometer were: number of fids, 65 536; number of scans, 128; probe temperature, 298 K; spectral width, 20.573 ppm; relaxation delay, 1.000 s; acquisition time, 4.819 s; pulse width, 90 °C; and total acquisition time, 16.0 min [6]. In addition, two-dimensional ^1^H-^1^H COSY and TOCSY spectra were acquired to assist the identification of particular aldehydic LOPs, together with other oil constituents. Both ^1^H-^1^H COSY and TOCSY spectra were programmed to acquire with no sweep width optimization and at 128 and 8 numbers of scans, respectively. 

A 0.60 mL volume of deuterochloroform (CDCl_3_) (99.8% purity) was added to a 0.30 mL aliquot of sampled culinary oils in a polypropylene vial. The solution was evenly mixed for not more than 10 s, after which a 0.50 mL aliquot was immediately pipetted into a 5 mm diameter NMR tube (Norrell HT, GPE Scientific, Leighton Buzzard, UK). Following this, samples were then made up to a final volume of 0.60 mL by the addition of 0.10 mL of a 1.10 mM solution of 1,3,5-tribromobenzene (TBB) in CDCl_3_. Indeed, CDCl_3_ served to provide a field frequency lock for the culinary oil samples, and TBB served as an internal quantitative ^1^H NMR standard for the determination of NMR-detectable autoxidation products in both control and thermally stressed oil samples. However, chemical shift values were referenced to tetramethylsilane (TMS) (δ = 0.000 ppm), residual CDCl_3_ (δ = 7.283 ppm) and/or TBB (δ = 7.537 ppm), each of which had the same starting concentrations for all ^1^H NMR measurements [5,6]. 

### 2.6. Analysis of ^1^H NMR Spectra

#### 2.6.1. Analysis of Major Lipid Species and IVs

**Figure 1:**
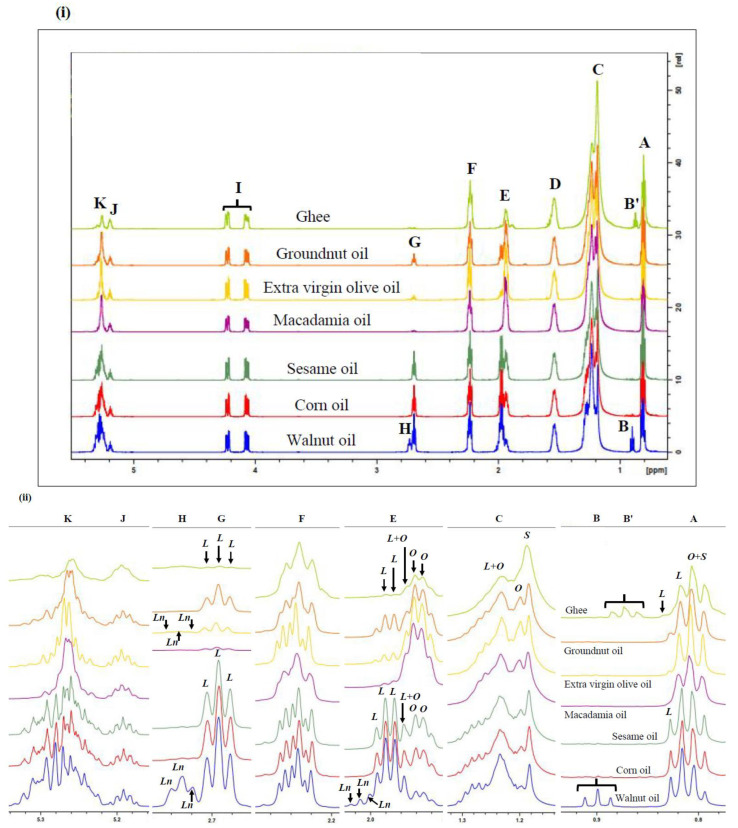
^1^H NMR spectra of (**i**) major acylglycerol function resonances present within the 0.0–5.4 ppm regions of unheated culinary oils. Letter assignments of resonances correspond to those provided in Table 2. ^1^H NMR spectra of (**ii**) expanded regions of resonances A, B, C, E, F, G, H, J and K present within the 0.0–5.4 ppm regions of unheated culinary oils. Abbreviations: *S*—saturated fatty acid acylglycerol signal; *O*—oleoylglycerol signal; *L*—linoleoylglycerol signal; *Ln*—linolenoylglycerol signal; *O*+*S*—composite oleoylglycerol and saturated fatty acid acylglycerol signal; *L*+*O*—composite linoleoylglycerol and oleoylglycerol fatty acid acylglycerol signal. Letter assignments of resonances correspond to those provided in Table 2.

Major compounds identified were predominantly those with selected key fatty acid acyl chains. The molar % of these acyl groups were deduced by inferring from their corresponding signal resonances in the ^1^H NMR spectra acquired on oil samples (Figure 1). The intensity of olefinic protons in these spectra was used to calculate and quantify IVs since an oil’s olefinic protons correlate linearly with the IV of that oil. The mathematical computations of the acyl groups and IV were performed according to the approaches employed by [12,14]. 

In addition, the molar % of *n*-butyric acid/ester acyl groups, which was only identified in ghee, was derived from Equation (1).

*n*-Butyric acid/ester acyl groups (molar %) = [100 − UFAs] − [100 × (1 − (*A*_E_/3 *A*_I_)] (1)

where *A*_E_ is the area of signal E (*mono*-allylic protons of all unsaturated acyl groups), *A*_I_ is the area of signal I (protons of carbon atoms 1 and 3 of the triacylglycerol glyceryl backbone), and UFAs is the overall derived molar % of all unsaturated acyl groups of the analyzed culinary oil.

Deductions of the key FA groups were computed from results acquired on three replicates, and the results are shown as mean ± standard deviation (SD) (molar % for acyl groups and unit for IV) [6].

#### 2.6.2. Analysis of Minor Compounds

The assignment of all minor compound resonances in the ^1^H NMR spectra acquired on oil samples were based on the review by [12]. The signal resonance of 1,2-diacylglycerols was assigned based on an extensive report of ^1^H and ^13^C NMR evaluations of encapsulated marine oil supplements [15]. The identification of these primary triacylglycerol hydrolysis products was performed for the characterization of the culinary oils analyzed [16,17,18].

#### 2.6.3. Analysis of LOPs

The assignment of the resonances of both primary and secondary LOPs identified in the ^1^H NMR spectra of the thermally stressed culinary oils were performed according to [3,6,12,16,17,18,19,20,21]. Amongst all the LOPs identified and characterized, only secondary or tertiary aldehydic LOPs were quantified. Aldehyde levels were computed for each of the three replicates, and these are presented as mean ± SD values (mM) [6]. 

In order to characterize the unidentified unsaturated aldehyde (UUA, signal k) detected in the PUFA-rich oils analyzed in this report, a 20 mL volume of PUFA-rich sunflower oil was thermally stressed continuously for 300 min, and a 0.30 mL aliquot was sampled and prepared, as described in Section 2.4 for ^1^H and ^1^H-^1^H TOCSY NMR analysis. This experiment was conducted on sunflower oil only in view of the greater thermo-oxidative susceptibility of sunflower oil [6] of all the oils examined in this report, in view of the greater possibility of detecting and identifying the rare UUA (signal l—as labeled in the report of [6].

### 2.7. Statistical Analysis of Experimental Data

An analysis of variance (ANOVA)-based experimental design was utilized to determine the significance of the ‘Between-Heating Method’ (i.e., continuous versus discontinuous), ‘Between Oil Classes’ and ‘Between-Thermal Stressing Time-Points’ effects incorporated into the study, together with those arising from the Heating Method × Oil Class, Heating Method × Thermal Stressing Time-Point, and Oil Class × Thermal Stressing Time-Point first-order interaction effects (Model 1). 

Hence, the overall experimental design for this Model 1 investigation was classified as a 3-factor system with the ‘Between-Heating Method’, ‘Between-Oil Class’ and ‘Between-Thermal Stressing Time-Point’ factors involved being fixed effects at 2, 7 and 11 classifications, respectively. Hence, this ANOVA model therefore comprised the 3 main fixed effect factors, their 3 associated first-order interactions and fundamental error (Equation (2)). In this equation, µ represents the null value in the absence of all sources of variation, and M*_i_*, O*_j_*, T*_j_*, MO*_ij_*, MT*_ik_*, OT*_jk_* and e*_ijkl_* represent the ‘Between-Heating Methods’, ‘Between-Oil Classes’, ‘Between-Thermal Stressing Time-Points’, and Heating Method × Oil Class, Heating Method × Thermal Stressing Time-Points, and Oil Class × Thermal Stressing Time-Point interaction effects, and unexplained error sources of variation, respectively.

y*_ijkl_* = µ + M*_i_* + O*_j_* + T*_k_* + MO*ij* + MT*ik* + OT*jk* + e*ijkl* (2)

Although Model 2 involved the application of ANOVA to the same fatty acid composition/IV and aldehydic LOP datasets, the number of main effect factors were limited to ‘Between-Heating Methods’ (i.e., continuous versus discontinuous thermal-stressing strategies for 120 min periods only) and Between-Oil Classes’, along with their first-order interaction effect (Equation (3)).

y*_ijk_* = µ + M*_i_* + O*_j_* + MO*ij* + e*ijk* (3)

## 3. Results

### 3.1. Characterisation of ^1^H NMR Spectra of Unheated Culinary Oils

#### Major Compounds Detectable

Resonances arising from the major acyl FA groups of ghee, groundnut, extra virgin olive, macadamia, sesame, corn and walnut oils present in their ^1^H NMR profiles are shown in Figure 1. This is supported by descriptions of their respective chemical shift values, multiplicities, and functional group assignments (Table 2). The assignments of ^1^H NMR signal resonances of each acyl group or function in the culinary oils evaluated follow those reported by [12,22,23].

The overlapping terminal methyl group proton (–C**H**_3_) resonances of all saturated, oleic and linoleic acid acyl chains in triacylglycerols are visible in the ^1^H NMR profiles acquired in view of their triplet multiplicities, i.e., the signal A composite located within the 0.780–0.839 ppm region (Figure 1i). This expanded spectral segment showed that the composite oleoylglycerol and saturated fatty acid acylglycerol signals (*O*+*S*) was the predominant one in SFA-rich oil (ghee) and MUFA-rich oils (groundnut, extra virgin olive and macadamia oils) (Figure 1ii). Conversely, the corresponding linoleoylglycerol resonance (*L*) was of a higher intensity than the *O*+*S* one in PUFA-rich oils (sesame, corn and walnut oils), as expected (Figure 1ii). 

**Figure 2:**
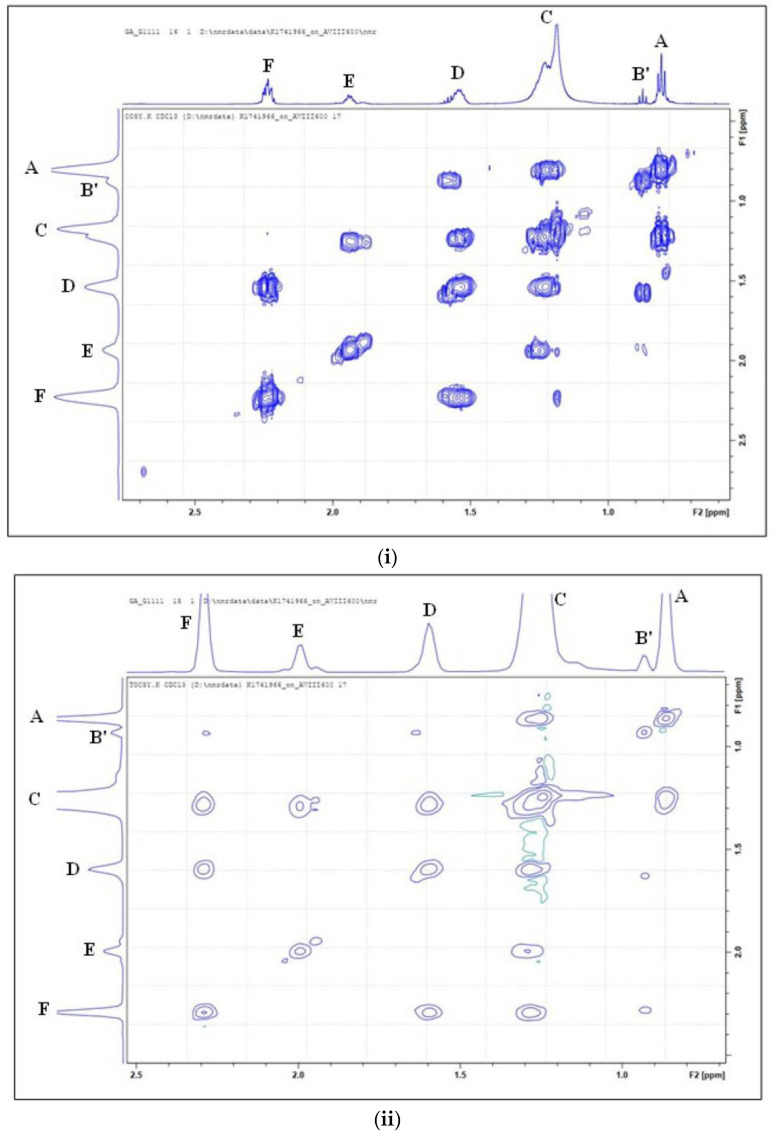

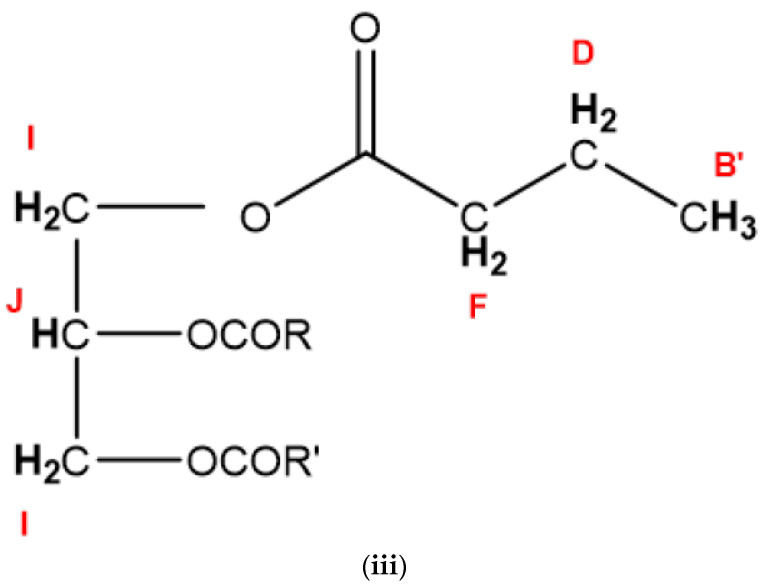
Two dimensional ^1^H–^1^H (**i**) COSY and (**ii**) TOCSY spectra of resonances A, B’, C, D, E and F present within the 0.0–2.5 ppm regions of unheated ghee. Letter assignments of resonances correspond to those provided in Table 2. (**iii**) An open structure of butyric acid/ester acyl groups identified in the ^1^H (Figure 1i,ii), as well as in the ^1^H-^1^H COSY (Figure 2i) and TOCSY (Figure 2ii) NMR spectra of ghee. R and R’ denote any FA acyl groups esterified to the triacylglycerol backbone. Linked ^1^H-^1^H resonances for the acyl fatty acid chains of SFAs, MUFAs and PUFAs are also clearly visible in these spectra.

Signals B’ and B are triplets positioned at 0.856–0.893 and 0.883–0.918 ppm and are ascribable to the terminal –C**H**_3_ group of *n*-butyric acid/ester and ω-3 FA acyl groups, respectively (Figure 1ii). Signal B’, which was exclusively detected in the ^1^H NMR spectra of ghee, was identified by 2D ^1^H-^1^H COSY and TOCSY analysis, as shown in Figure 2i,ii, respectively. In contrast, signal B was only identified in walnut oil and was ^1^H NMR-undetectable in groundnut, extra virgin olive, macadamia, sesame and corn oils, as expected (Figure 1ii). The difference between the δ values of signals A and B is a reflection of the terminal –CH_3_ group’s proximity to the nearest >CH=CH< double bond function in linoleoyl- and linolenoylglycerols, respectively.

In Figure 2iii, the ^1^H nuclei attached to carbon atoms 4, 3 and 2 (nomenclature referenced to the carbon atom 1 in –O**C**O– of butyric acid/ester) are represented as signals B’, D and F in the ^1^H (Figure 1i), COSY (Figure 2i) and TOCSY (Figure 2ii) spectra. In addition, the protons in the triacylglycerol backbone of FA acyl groups characterized in Figure 1i are also represented in Figure 2iii. To show which pairs of ^1^H nuclei in butyric acid/ester acyl groups are coupled to each other, the COSY spectrum confirms that signal B’ is coupled to signal D, which in turn is also coupled to signal F (Figure 2). This was confirmed in the TOCSY spectrum by the identification of all ^1^H nuclei that belonged to the same coupled spin system, i.e., signals D and F were the cross-peaks of signal B’.

Signal C, which represents a composite series of superimposing multiplets within the 1.119–1.344 ppm region, is ascribable to the bulk chain methylene protons (–(C**H**_2_)*n*–) of FA acyl groups (Figure 1i). When expanded, signal C comprised resonances assignable to saturated fatty acid acylglycerols (*S*), oleoylglycerols (*O*) and composite linoleoylglycerols and oleoylglycerols (*L*+*O*) (Figure 1ii). The intensity of the *L*+*O* resonance was found to be inversely proportional to that of *S*, and this was largely dependent on the saturation degree of the oil (Figure 1ii). This observation served to distinguish between the studied SFA-, MUFA- and PUFA-rich culinary oils. In retrospect, the highest intensity of *S* and the lowest intensity of the *L*+*O* signals were found in the ghee sample (Figure 1ii). Resonances attributable to *O* were sparingly detectable in the ghee sample. However, resonances attributable to *O* were easily identified in all the other culinary oils, with their intensity being higher and lower in MUFA- and PUFA-rich oils, respectively (Figure 1ii).

Signal D is a multiplet located in the 1.494–1.596 ppm range, which arises from the β-position methylenic protons (–OCO–CH_2_–C**H**_2_–) of all acyl groups present in the oils (Figure 1i). 

Signal E is attributable to the *mono*-allylic protons (–C**H**_2_–CH=CH–) of all acyl groups (Figure 1i). When expanded, this signal comprises contributions from linolenoylglycerols (*Ln*), along with *L* and *O* (Figure 1ii). *Ln* was found to be exclusive to walnut, extra virgin olive and macadamia oils (Figure 1ii). However, the intensity of *Ln* was much lower in extra virgin olive and macadamia oils (Figure 1ii). Ghee was found to have the lowest intensity for all signals assignable to *L* (Figure 1ii). Similarly, the *L* and *O* components of signal E in spectra acquired on macadamia oil were of the lowest and the highest intensities, respectively (Figure 1ii). Of course, the intensities of the *L*, *O* and *L*+*O* signals served to distinguish between the SFA-, MUFA- and PUFA-rich oils. Indeed, the unsaturation degree of culinary oils was directly proportional to the intensities of the *L* and *Ln* and inversely proportional to the intensities of the *O* signals (Figure 1ii).

Signal F (2.200–2.286 ppm) is attributable to the α-protons (–OCO–C**H**_2_–) of all acyl groups (Figure 1i). Amongst all the oils investigated, this signal was distinct in the ^1^H NMR profiles of ghee, groundnut and extra virgin olive oils. Likewise, this observation was similar to that made in the ^1^H NMR profiles of sesame, corn, and walnut oils (Figure 1ii). 

Signals G and H are both characterized by doublet-of-doublet multiplicities. These resonances are ascribable to the *bis*-allylic protons (=HC–C**H**_2_–CH=) of ω-6 diunsaturated and ω-3 triunsaturated FAs, respectively. Signal H is exclusive to linolenic acid acyl groups (Figure 1ii), and as expected, it was ^1^H NMR-undetectable in ghee, groundnut, sesame and corn oils, in view of their very low contents of *Ln* (Figure 1ii). Signals G and H are, therefore, key features required for the quantification of linoleic and linolenic acid triacylglycerols (Figure 1ii). As expected, reductions in the intensities of signals G and H served as reflective indicators of the thermo-oxidative susceptibility of the studied culinary oils.

Signal I arises from the protons on carbon atoms 1 and 3 of the glyceryl backbone functions of acyl groups (–C**H**_2_OCOR). Characterized as an ABX (AB) coupling pattern, signal I was positioned at 4.032–4.266 ppm in the ^1^H NMR profiles acquired (Figure 1i). Similarly, signal J (δ = 5.164–5.221 ppm), which is of ABX (X) coupling pattern, is attributable to the protons on carbon atom 2 of the glyceryl backbone unit (Figure 1i). The overall intensities of signals I (Figure 1i) and J (Figure 1ii) were similar among all the culinary oils investigated.

Signal K is a multiplet assignable to the olefinic functions (–C**H**=C**H**–) of several acyl groups. The difference in the unsaturation degree of the culinary oils, however, led to a considerable variation in the δ-scale values and intensities of this resonance amongst the oils studied here, however (Figure 1). When expanded, the multiplet nature of signal K became more complex and in direct proportion to the oil’s higher unsaturation degree. This explained the disparities of signal K amongst SFA-rich ghee, MUFA-rich groundnut, extra virgin olive and macadamia oils, and PUFA-rich sesame, corn and walnut oils (Figure 1ii). Of course, signal K is central in determining the total unsaturation degree, which is also represented by the IV of the oils evaluated. 

In addition, the 600 MHz frequency ^1^H NMR spectroscopy also allowed for the detection of minor compounds in the studied culinary oils. These are discussed in the Supplementary Information Section 1.1. The resonances of all ^1^H NMR detectable minor compounds are also profiled and described in Supplementary Materials Figure S1 and Table S1, respectively.

It is, however, worth noting that there is a controversy in the literature on the identification of signal B’. For example, [24] reported that this resonance identified in fat extracts of hard and white fermented cheese is ascribable to short-chain FAs of 4–8 carbon atom lengths, although an 8 carbon FA chain may be classified as a medium-chain FA. Additionally, the publication by [25] demonstrated that with the aid of a 300 MHz NMR spectrometer, the 6 carbon chain FA, caproic acid, has its terminal –C**H**_3_ resonance shifted up-field and hence contributes significantly to signal A. Nonetheless, as analyzed on a higher resolution NMR spectrometer, this present study established the contested ^1^H NMR signal B’ as resonances of *n*-butyric acid, and this was confirmed by the application of ^1^H-^1^H COSY and TOCSY techniques. This is in agreement with a recent publication by [26], who characterized the same signal B’ in milk fats as *n*-butyric acid. As a merit, this can be used as a ^1^H NMR marker of milk TAGs, in complement to the ^13^C study originally published by [27].

### 3.2. Oxidation Products of UFAs

The thermal degradation of UFAs of culinary oil at higher temperature ranges (70–190 °C) is accompanied by the evolution of LOPs with varying degrees of chemical reactivities [5,6,8,17,18,28,29,30].

#### 3.2.1. Primary LOPs

All ^1^H NMR detectable LOPs identified and characterized in the studied culinary oils are categorized as either primary or secondary LOPs. Primary LOPs, such as conjugated hydroperoxydienes (CHPDs), are profiled in the Supplementary Information Figures S2 and S3, and the descriptions of all the identified resonances are provided in Table S2. These are also discussed in the Supplementary Materials Section 1.2.1.

#### 3.2.2. Secondary LOPs

Secondary LOPs are relatively stable, albeit potentially harmful chemical species that may pose a greater risk to healthy cells [1,21]. Secondary LOPs are derived from primary LOPs, and these include epoxy-FAs, primary alcohols, and aldehydes, amongst many other classes of degradation products.

##### Epoxy-FAs and Primary Alcohols

Epoxy-FAs and primary alcohols identified in the thermo-oxidized culinary oils are discussed in the Supplementary information Section 1.2.2.1. All identified epoxy-FAs and primary alcohols are listed in Table S3, in the Supplementary Materials and their respective resonances are also shown in the ^1^H NMR profiles of Figure S4. 

##### Aldehydes

Aldehydic LOPs identified in thermally stressed culinary oils were categorized as either saturated aldehydes or α,β-UAs. The presence of diene, conjugated diene and/or hydroxy-/hydroperoxy functions in selected α,β-UAs renders them more reactive than saturated aldehydes. This increased reactivity underscores the greater toxicity of α,β-UA LOPs [1,31,32]. 

**Figure 3:**
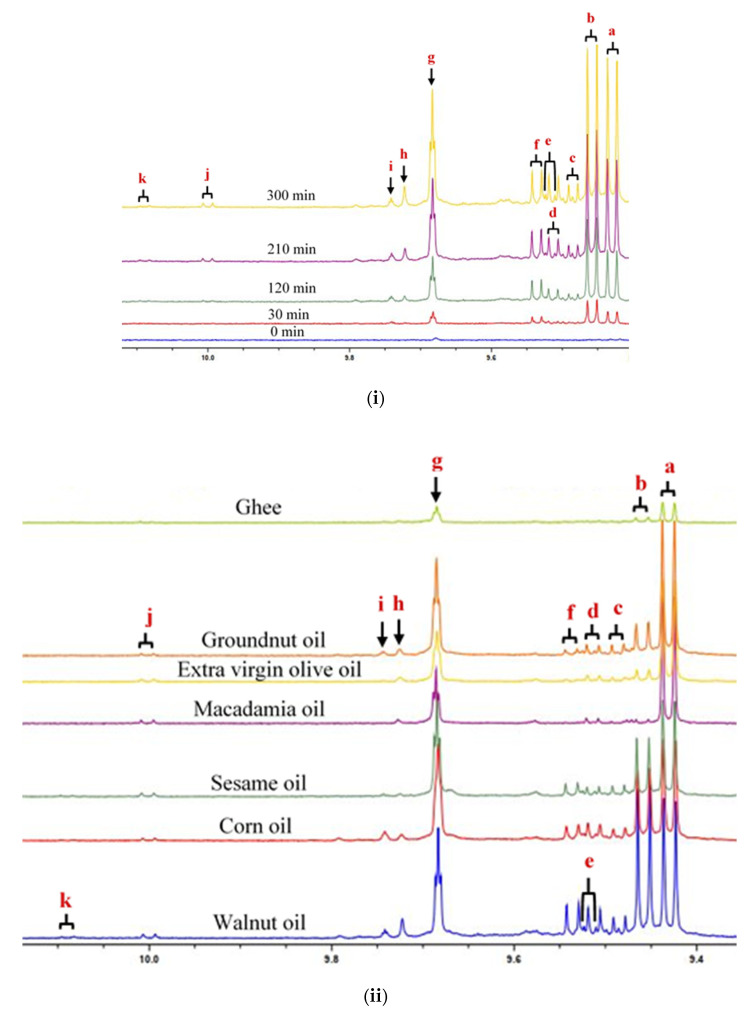
^1^H NMR profiles showing expanded aldehyde-CHO function (9.40–10.20) ppm regions of (**i**) walnut oil thermally stressed for increasing durations and (**ii**) all culinary oils thermally stressed for an extreme 300 min period. Letter assignments of resonances correspond to those provided in Table 3.

The α,β-UAs aldehydes detected in the thermally stressed culinary oils comprised (*E*)-2-alkenals (signal a), (*E,E*)-2,4-alkadienals (signal b), 4,5-epoxy-(*E*)-alkenals (signal c), 4-hydroxy-(*E*)-2-alkenals (signal d), 4-hydroperoxy-(*E*)-2-alkenals (signal e), (*Z,E*)-2,4-alkadienals (signal f), and (*Z*)-2-alkenals (signal j). Being a doublet, the unidentified unsaturated aldehyde (UUA) resonance (signal k), which has a *J* value of 7.96 Hz, is potentially assignable to another α,β-UA (Figure 3). There is also a significant overlap of the signal resonance of 4-hydroxy-(*E*)-2-alkenals (signal d) with that of 4-hydroperoxy-(*E*)-2-alkenals (signal e) (Figure 3). *n*-Alkanals (signal g), 4-oxo-*n*-alkanals (signal h) and *n*-alkanals of low molecular mass (ethanal, propanal and butanal, etc.) (signal i) were three classes of ^1^H NMR-distinguishable saturated aldehydes in the oil samples analyzed (Figure 3). 

Prolonged thermo-oxidation of culinary oils at a constant temperature of 180 °C resulted in incremental changes in the levels of aldehydic LOPs detectable therein. Although this was profiled for all oils evaluated, this is depicted in Figure 3i for the 300 min continuous thermo-oxidized walnut oil. The evolution of the differential types of LOPs, as well as their concentrations in the thermally stressed culinary oils, were dependent on the oils’ unsaturation degree, as expected. However, PUFA-rich culinary oils (sesame, corn and walnut oils) produced the largest amounts of all ^1^H NMR-detectable LOPs and also contained a broader spectrum of such LOP classes than those produced in MUFA-rich oils and SFA-rich ghee (Figure 3ii). It should also be noted that the intensities of aldehydic LOP resonances were the lowest in thermo-oxidized ghee (Figure 3ii), and these were attributable to only three types of aldehydes, specifically (*E*)-2-alkenals, *n*-alkanals and 4-oxo-*n*-alkanals. These secondary LOPs were also characterized in SFA-rich coconut oil, which was thermally stressed according to both continuous and discontinuous protocols without any partial oil substitutions being conducted [6], in addition to those observed in a continuous manner with such substitutions [18], under similarly controlled experimental conditions. 

In general, the concentration magnitudes of these secondary LOPs were found to be in the order walnut > corn > sesame > groundnut > extra virgin olive > macadamia oils > ghee (Figure 3ii). Aldehydic LOPs identified in this report are fully consistent with those reported by [5,6,8,16,17,18,20,21,33,34]. 

**Figure 4:**
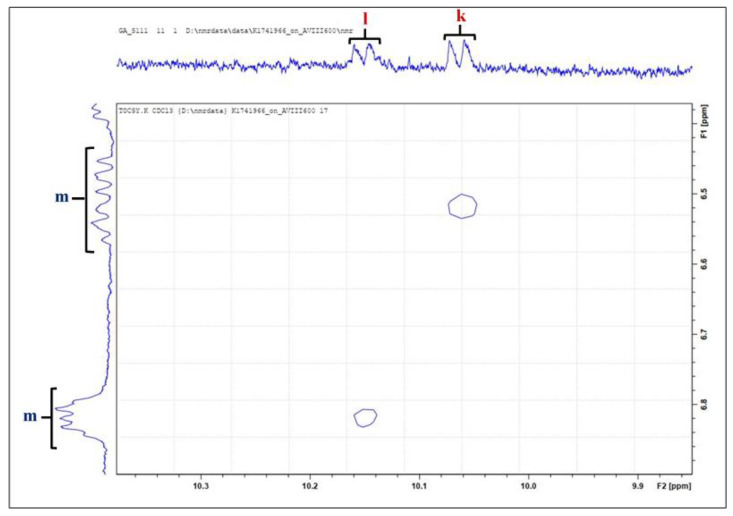
Two dimensional ^1^H–^1^H TOCSY spectrum of resonances UUA (signal k) and (*Z*)-2-alkenals present within the 10.00–10.20 ppm regions of 600 MHz ^1^H NMR spectra of thermo-oxidized sunflower oil. Letter assignments of resonances correspond to those provided in Table 3 and Supplementary Information Table S2.

In Figure 4, the cross-peak of signal j reveals direct coupling to the olefinic protons of signal m (δ = 6.442–6.583 ppm), and this assignment was first reported by [21] as (*Z*)-2-alkenals species. The cross-peak of signal k demonstrates coupling to the olefinic protons of multiplet signal m (δ = 6.778–6.866 ppm *J* = 8.13 Hz). An analysis of the cross-peaks identified in the TOCSY spectrum of sunflower oil for the aldehydes (9.40–10.20 ppm) and their olefinic protons (5.50–7.00 ppm) illustrates that signal k is undoubtedly an alkenal species with its –CH=CH– unit in the β- and γ-positions. This association of signal k with the olefinic proton located at δ = 6.778–6.866 ppm is also provided in Table S2 of the Supplementary Materials.

## 4. Discussion

### 4.1. Culinary oil FA Compositions and IVs

**Figure 5:**
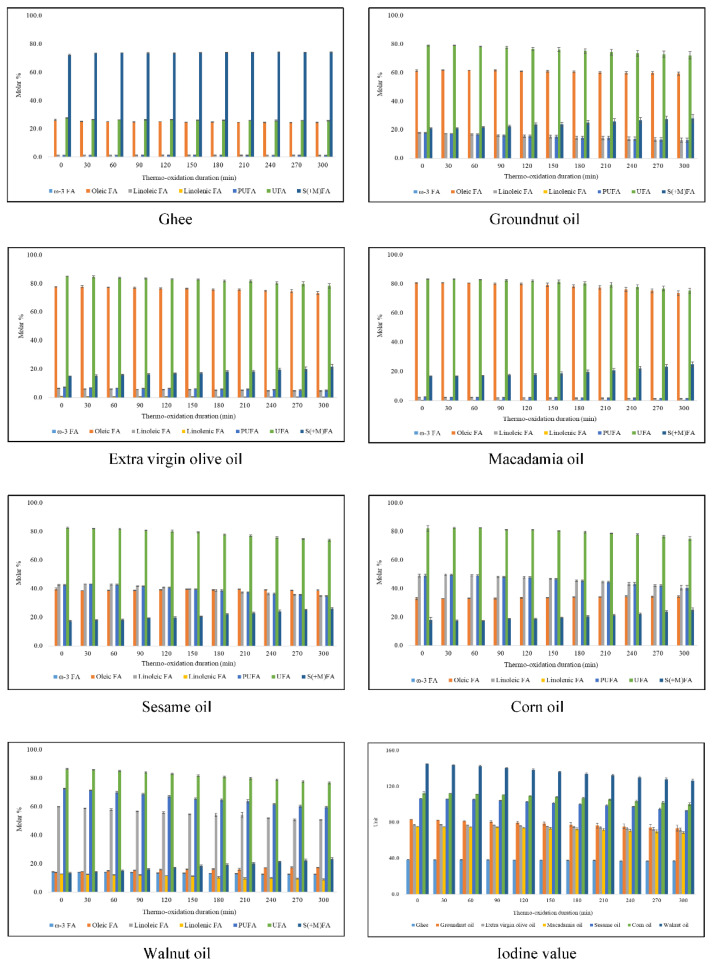
Changes in acyl groups and iodine values of thermally stressed culinary oils subjected to a continuous thermo-oxidation at 180 °C for 300 min. The values are presented as mean ± SD molar % (FA) and mean ± SD unit (IV) of oil.

**Figure 6:**
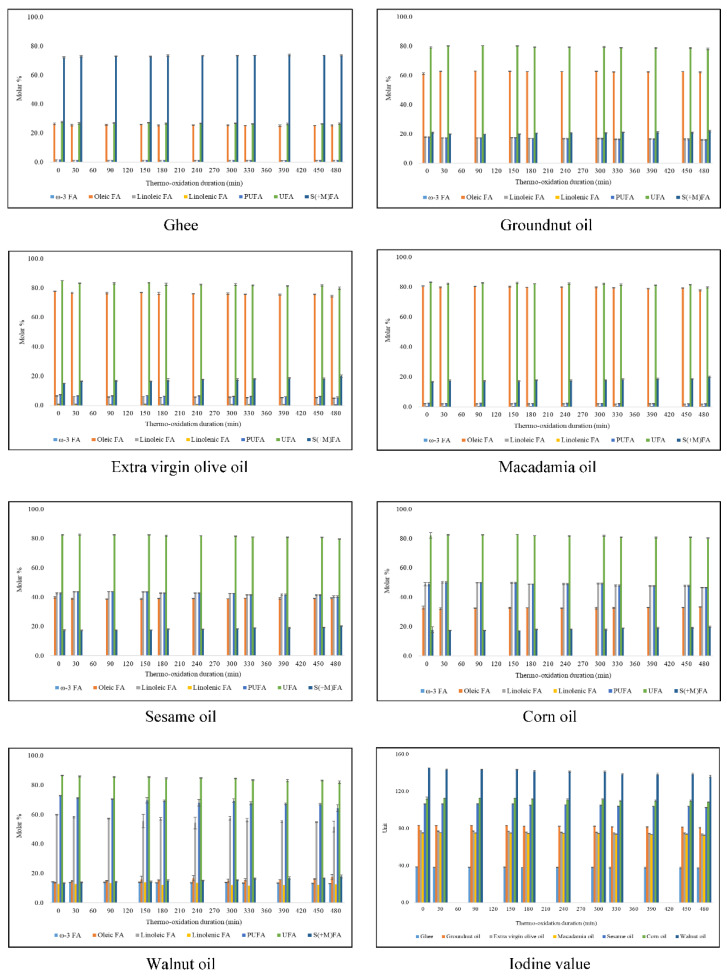
Changes in acyl groups and iodine values of thermally stressed culinary oils subjected to a discontinuous thermo-oxidation at 180 °C for 480 min. The values are presented as mean ± SD molar % (FA) and mean ± SD unit (IV) of oil.

Modifications in acyl group contents observed when exposed to thermal-stressing episodes at 180 °C are fundamental for the explanation of the peroxidative susceptibilities of culinary oils during thermo-oxidation processes. The thermo-induced changes in these FA contents during both continuous and discontinuous thermo-oxidation of culinary oils are available in Figure 5 and Figure 6, respectively. Thermo-oxidation of culinary oils resulted in reductions in UFA levels, as expected, and the concomitant increase in their saturated (and modified) fatty acid concentrations (S(+M)FAs), along with the evolution of LOPs. The molar % of linoleic and linolenic FAs (present in extra virgin olive, macadamia and walnut oils) and total PUFA and UFA contents decreased appreciably during the 300 min duration with continuous thermo-oxidation. Consistently, gains in S(+M)FAs levels of the oils were proportional to losses in their UFA contents (Figure 5). When subjected to discontinuous thermo-oxidation episodes, the modifications in linoleic and linolenic acids, and total PUFAs and UFAs, were, however, less profound. This is attributed to the 120 min regular cooling effect implemented after every 30 min of thermo-oxidation during the discontinuous heating episodes conducted (Figure 6). 

During continuous (300 min) and discontinuous (480 min) thermo-oxidation episodes for each culinary oil investigated, the oleic acid contents increased from 13.7 to 17.2 and 17.6 molar %, respectively, in PUFA-rich walnut oil, a consequence of diminishing PUFA levels. However, smaller changes in oleic acid contents were recorded in sesame (39–40 molar %) and corn oils (32–35 molar %) during continuous and discontinuous thermo-oxidation episodes, respectively (Figure 5 and Figure 6). Similarly, smaller changes in oleic acid contents were recorded in ghee (25–26 molar %) and groundnut oil (61–63 molar %) when they were thermally stressed discontinuously for a 480 min duration (Figure 6). Furthermore, oleic acid levels decreased from 26.3 to 24.6, from 61.2 to 59.3, from 77.7 to 73.2 and from 80.6 to 73.7 molar % continuously in thermally stressed ghee, groundnut, extra virgin olive and macadamia oils, respectively (Figure 5). Likewise, the discontinuous thermo-oxidation of extra virgin olive and macadamia oils gave rise to a decrease in oleic acid contents from 77.7 to 74.4 and from 80.6 to 77.7 molar %, respectively (Figure 6). The smaller changes observed in oleic acid contents reflect their relative resistance to thermo-oxidation during these episodes [6].

**Figure 7:**
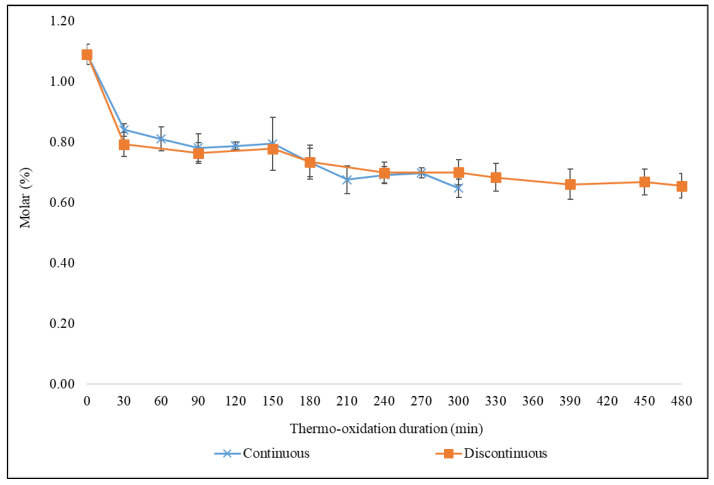
Changes in butyric acid/ester acyl groups of ghee subjected to a continuous and discontinuous thermo-oxidation at 180 °C for 300 and 480 min, respectively. The values are presented as mean ± SD molar % butyric acid/ester acyl groups of ghee.

The increases in oleic acid and S(+M)FAs contents observed in the oils tested predominantly arise from decreases in the most highly peroxidatively susceptible FAs present therein; specifically, via time-dependent decreases in overall PUFA and ω-3 FA contents results in a proportionate increase in the above FAs, which have less susceptibility to peroxidation. Interestingly, the two thermo-oxidative processes employed resulted in reductions in the concentration of butyric acid and its ester derivatives (Figure 7). Indeed, this is an indication that the thermally induced increase in S(+M)FAs is predominantly accounted for by the decreasing contents of UFAs observed.

As noted above, ω-3 FAs were only ^1^H NMR-detectable in walnut oil, and regardless of the thermo-oxidation episodes employed, these decreased from 14.3 molar % in unheated walnut oil to 12.5 and 13.1 molar % in continuous and discontinuous thermo-oxidized walnut oils, respectively (Figure 5 and Figure 6). By comparison, ω-3 FAs decreased by 1.8 and 1.2 molar % in continuous and discontinuous thermo-oxidized walnut oil, respectively, and these were not dissimilar to the 2.5 and 1.3 molar % evaluated by [6] in continuous and discontinuous thermo-oxidized rapeseed oil, respectively, despite there being a 4.2-fold lower molar % of ω-3 FAs in unheated rapeseed oil [6].

The FA content changes observed at a constant temperature of 180 °C over a defined thermo-oxidation duration are mainly ascribable to differences in the degree of unsaturation between the culinary oils. Overall, thermo-oxidation of UFAs and the molar % gain of S(+M)FAs for a 300 min in a continuous manner gave results that were in the order walnut (9.8 molar %) > sesame (8.7 molar %) > macadamia (8.0 molar %) > corn (7.1 molar %) > groundnut (7.0 molar %) > extra virgin olive oils (6.7 molar %) > ghee (1.9 molar %) (Figure 5). 

Likewise, the thermo-oxidation of UFAs throughout the 480 min discontinuous thermo-oxidation episode for culinary oils was in the order extra virgin olive (5.1 molar %) > walnut (4.5 molar %) > macadamia (3.4 molar %) > sesame (2.8 molar %) > corn oils (2.0 molar %) > ghee (1.2 molar %) > groundnut oil (1.0 molar %), and these values were of the same magnitude as the molar % gain of S(+M)FAs groups (Figure 6). For a longer thermo-oxidation duration at a constant temperature, it is therefore evident that changes in the degree of unsaturation of culinary oils are impacted by the type of thermo-oxidation episodes employed.

At the 300 min time-point for continuous thermo-oxidation, IVs decreased in the order; walnut (18.7 units) > sesame (13.3 units) > corn (12.4 units) > groundnut (9.8 units) > macadamia (6.9 units) > extra virgin olive oils (5.6 units) > ghee (1.6 units) (Figure 5). However, for the total 480 min discontinuous thermo-oxidation duration, IVs decreased in the order walnut (9.2 units) > corn (4.1 units) > extra-virgin olive (4.1 units) > sesame (3.9 units) > groundnut (2.7 units) > macadamia oil (2.6 units) > ghee (1.3 units) (Figure 6). In both thermo-oxidation episodes, the loss of unsaturation degree was the most severe for PUFA-rich walnut oil and least so for SFA-rich ghee. Furthermore, discontinuous thermo-oxidation episodes led to a preservation of unsaturation degree more so than that observed during continuous thermo-oxidation. This observation supports the conclusions made by [6]. 

**Figure 8:**
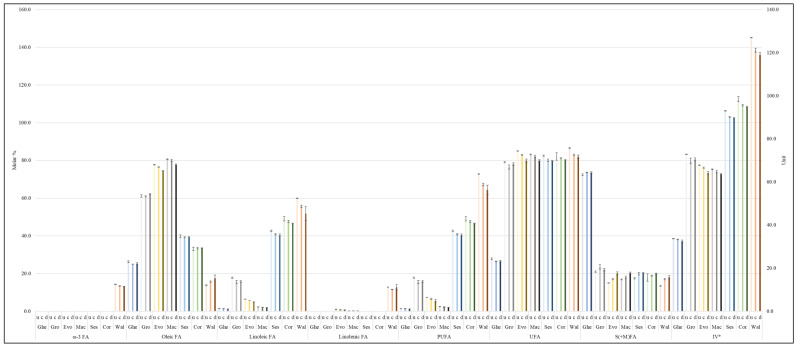
Modifications to FA groups and IVs in unheated and thermally stressed culinary oils exposed to continuous and discontinuous thermo-oxidation episodes conducted at 180 °C for 120 min. IV* is expressed in IV units. Abbreviations: u—unheated oil; c—continuous thermo-oxidation episode; d—discontinuous thermo-oxidation episode; Ghe—ghee; Gro—groundnut oil; Evo—extra virgin olive oil; Mac—macadamia oil; Ses—sesame oil; Cor—corn oil; Wal—walnut oil; ω-3 FA—omega-3 fatty acid; PUFA—polyunsaturated fatty acid; UFA—unsaturated fatty acid; S(+M)FA—saturated (and modified) fatty acid; IV—iodine value. The values are presented as mean ± SD molar % (FA) and mean ± SD IV units of each oil tested.

In comparison, there were small changes in the FA contents and IVs between the 120 min time-point continuous and discontinuous thermo-oxidation episodes (Figure 8). Oleoylglycerols were higher in all culinary oils, except extra virgin olive and macadamia oils, which were thermally stressed discontinuously for 120 min. In contrast, linoleoylglycerols were higher in 120 min discontinuous thermo-oxidized oils, except for groundnut oil, which had higher linoleoylglycerols at the 120 min time-point of continuous thermal stressing episodes (Figure 8). The oxidation of oleoylglycerols and linoleoylglycerols mostly generate saturated aldehydes, as well as unsaturated ones such as (*E*)-none-, dece- and undecenals [35]. 

Butyric acid and its ester derivatives (Figure 7) and ω-3 acyl groups (Figure 8), which were only ^1^H NMR-detectable in ghee and walnut oil, respectively, were also higher at the 120 min continuous thermal stressing time-point than they were at the corresponding discontinuous thermo-oxidation one. The higher formation of butyric acid/ester groups stems from the modifications of several FA acyl groups with similar properties as butyric acid/ester groups. Additionally, higher levels of ω-3 acyl groups at 120 min of continuously thermo-oxidized walnut oil are an indication of how the repeated use of reheated oils, as in discontinuous thermo-oxidation, is overall damaging to UFAs (Figure 8).

The linolenoylglycerol content of walnut oil was lower at the 120 min continuous thermal stressing time-point than they were at the corresponding discontinuous thermo-oxidation phase (Figure 8). The oxidation of linolenoylglycerols produces complex aldehydes such as α,β-UAs [35], and these may influence their overall distribution at the 120 min discontinuous phase. Overall, at 120 min time-point, the discontinuous thermo-oxidation process decreased the molar % content of UFAs and IVs whilst simultaneously increasing the molar % of S(+M)FAs in all studied oils. This observed trend, however, was not applicable to the IVs, and UFA and S(+M)FA contents of ghee and groundnut oil evaluated at the 120 min time-point for both thermo-oxidation processes (Figure 8). Due to the nature of results obtained, which were also consistent with those reported by [6], future studies will, therefore, endeavor to study individual models of FA acyl groups to understand their dynamics in response to specific temperatures. 

### 4.2. Evolution of LOPs

**Figure 9:**
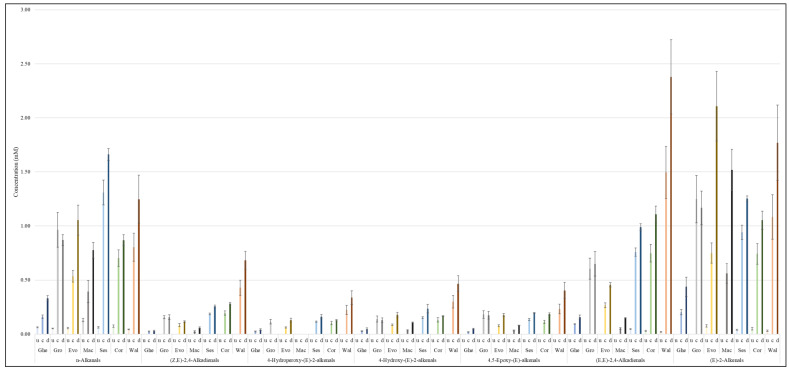
Evolutions of aldehydic LOPs (major types) in unheated and thermally stressed culinary oils exposed to continuous and discontinuous thermo-oxidation episodes at 180 °C for a 120 min period. Abbreviations: u—unheated oil; c—continuous thermo-oxidation episode; d—discontinuous thermo-oxidation episode; Ghe—ghee; Gro—groundnut oil; Evo—extra virgin olive oil; Mac—macadamia oil; Ses—sesame oil; Cor—corn oil; Wal—walnut oil. The values are presented as mean ± SD mM (mmol LOPs per 1.0 L volume of oil).

^1^H NMR analysis of even unheated culinary oils revealed the presence of low levels of LOPs in these products. Indeed, concentrations of 0.06 ± 0.00 mM *n*-alkanals were quantified in ghee; 0.05 ± 0.00 mM *n*-alkanals in groundnut oil; 0.08 ± 0.01 mM (*E*)-2-alkenals and 0.06 ± 0.01 mM *n*-alkanals in extra-virgin olive oil; 0.13 ± 0.02 mM *n*-alkanals in macadamia oil; 0.04 ± 0.00 mM (*E*)-2-alkenals, 0.05 ± 0.00 mM (*E,E*)-2,4-alkadienals, and 0.06 ± 0.01 mM *n*-alkanals in sesame oil; 0.05 ± 0.01 mM (*E*)-2-alkenals, 0.03 ± 0.01 mM (*E,E*)-2,4-alkadienals, and 0.07 ± 0.01 mM *n*-alkanals in corn oil; and 0.03 ± 0.01 mM (*E*)-2-alkenals, 0.02 ± 0.00 mM (*E,E*)-2,4-alkadienals, and 0.04 ± 0.01 mM *n*-alkanals were found in walnut oil (Figure 9, Supplementary Information Figures S6 and S7). 

The detection of these LOPs in unheated culinary oils presents a potential health hazard to consumers, although these levels are very small when compared to those found in samples that were exposed to high frying temperatures for increasing time periods. Previously, [6,18] found 0.09 ± 0.01 mM (*E*)-2-alkenals, 0.02 ± 0.00 mM (*E,E*)-2,4-alkadienals, and 0.09 ± 0.00 mM *n*-alkanals in unheated olive oil. However, higher concentrations of (*E*)-2-alkenals (0.15 ± 00 mM) and *n*-alkanals (0.15 ± 0.01 mM) were determined in unheated sunflower oil [6,18]. Nevertheless, no ^1^H NMR- detectable LOPs were identified in coconut and rapeseed oils [6,18]. LOPs in unheated oils have also been reported by [5]. The presence of LOPs in unheated oils has been attributed to possible exposure of these products to peroxidation-promoting sunlight and/or to prolonged storage of these products at above ambient temperatures [1,5,35].

Between the two thermo-oxidation episodes studied, the 300 min continuous thermo-oxidation periods resulted in higher production of LOPs than that measured in the 480 min discontinuous thermo-oxidation episodes involved (Supplementary Information, Figures S6 and S7). Conversely, the concentrations of α,β-UAs and low-molecular-mass *n*-alkanals on completion of the thermo-oxidation duration were higher in extra virgin olive oil undergoing discontinuous than they were during continuous thermo-oxidation episodes. Likewise, at the end of the thermo-oxidation duration, (*E*,*E*)-2,4-alkadienals, (*Z*,*E*)-2,4-alkadienals and (*Z*)-2-alkenals were also higher in the discontinuous thermo-oxidized macadamia oil than they were during the continuous process (Supplementary Information, Figures S6 and S7). For both macadamia and extra-virgin olive oils, (*Z*,*E*)-2,4-alkadienals were also present at higher levels throughout the discontinuous thermo-oxidation session than they were throughout the continuous one. Oxygen levels rejuvenate in the culinary oils during the cooling periods in the discontinuous thermo-oxidation phase. This dissolved oxygen contributes to the formation of LOPs in the oils despite the gradual continuous drop in temperature during the cooling of oils during the discontinuous thermo-oxidation phase. However, the incongruous data observed for the above LOPs detectable in macadamia and extra-virgin olive oils for the above-mentioned LOPs require further investigations to understand the factors contributing toward this observation.

For both the 300 min continuous and 480 min discontinuous thermo-oxidation episodes, PUFA-rich walnut oil produced the largest amounts of (*E*,*E*)-2,4-alkadienals, 4,5-epoxy-(*E*)-2-alkenals, 4-hydroxy-(*E*)-2-alkenals, 4-hydroperoxy-(*E*)-2-alkenals, (*Z*,*E*)-2,4-alkadienals, 4-oxo-*n*-alkanals and the alkenal species (signal k) (Supplementary Materials Figures S6 and S7). However, (*E*)-2-alkenals and low-molecular-mass *n*-alkanals were predominant at the 300 min continuous thermal stressing time-point for groundnut and corn oils, respectively. In addition, sesame oil exposed to the continuous thermo-oxidation protocol was found to contain the highest concentrations of *n*-alkanals and (*Z*)-2-alkenals (Supplementary Materials, Figure S6). Similarly, the discontinuous thermo-oxidation of sesame oil generated the highest level of *n*-alkanals. (*E*)-2-Alkenals and low-molecular-mass *n*-alkanals were found to be the most predominant aldehydes present in extra virgin olive and walnut oils thermally stressed discontinuously for a 480 min duration (Supplementary Materials, Figure S7). 

As expected, PUFA-rich sesame, corn and walnut oils were the most susceptible to thermo-oxidation at 180 °C and, therefore, produced the largest amounts of LOPs. In contrast, SFA-rich ghee produced the smallest levels (Supplementary Materials Figures S6 and S7). The study conducted by Le Gresley et al. (2019) also showed that SFA-rich coconut oil was the most resistant to thermo-oxidation at 180 °C and produced only three types of LOPs, which were (*E*)-2-alkenals; *n*-alkanals and 4-oxo-*n*-alkanals. However, as expected, MUFA-rich olive and rapeseed oils produced higher levels of aldehydic LOPs, which in view of their higher PUFA contents, were somewhat more class-variable than those generated by coconut oil. Additionally, PUFA rich-sunflower oil, the most peroxidatively susceptible culinary oil, yielded significant levels of the UUA (signal l in their report), along with all other classes of aldehydic LOPs identifiable by ^1^H NMR analysis, and at the highest concentrations found [6].

In continuous thermo-oxidation episodes, the thermo-oxidative resistance of MUFA-rich macadamia oil was clearly apparent and overall was similar to that observed for SFA-rich ghee. Except for (*E*)-2-alkenals, *n*-alkanals and (*Z*)-2-alkenals, which were found to be a little higher in macadamia oil than in ghee, the concentrations of the other aldehydic LOPs, i.e., those derived from MUFA peroxidation, were present at similar concentrations. Furthermore, it should be noted that low-molecular-mass *n*-alkanals detectable in ghee were undetectable in macadamia oil (Supplementary Materials, Figure S6). A similar trend was also observed for both oils under discontinuous thermo-oxidation episodes (Supplementary Materials, Figure S7). MUFA-rich macadamia oil, according to ^1^H NMR data presented here, therefore exhibits a thermo-oxidative resistance property that is similar to that of SFA-rich ghee. Therefore, the blending of this oil with PUFA-rich products is likely to limit thermo-oxidative damage within frying media throughout the course of their frying use. 

Another observation made here was the generation of some aldehydic LOPs only at the later thermo-oxidation durations. For example, 4,5-epoxy-(*E*)-2-alkenals only evolved at the 60 and 90 min time-points of continuous thermal stressing episodes for ghee and macadamia oil, respectively (Supplementary Materials, Figure S6). Moreover, with the discontinuous thermo-oxidation of ghee and macadamia oil, the first detection of 4,5-epoxy-(*E*)-2-alkenals was at the 180 and 240 min time-points, respectively (Supplementary Materials, Figure S7). Other aldehydic LOPs that evolved at these later time-points in ghee and macadamia oil subjected to both continuous and discontinuous thermo-oxidation episodes were 4-hydroxy-(*E*)-2-alkenals, 4-hydroperoxy-(*E*)-2-alkenals, (*Z*,*E*)-2,4-alkadienals, 4-oxo-*n*-alkanals, low-molecular-mass *n*-alkanals, (*Z*)-2-alkenals and the alkenal species (signal k) (Supplementary Materials, Figures S6 and S7). More importantly, the proportional distribution of aldehydic LOPs varied greatly between continuous and discontinuous thermo-oxidation episodes; these conclusions were previously made by Le Gresley et al. (2019).

As noted above, the formation of secondary LOPs is preceded by the formation of lipid hydroperoxides, which are primary LOPs. Indeed, CHPDs (from PUFA peroxidation) have two >CH=CH< functions in the same molecule, but hydroperoxymonoenes (HPMs) (from MUFA peroxidation) only have a single >CH=CH< unit. Although more structurally complex aldehydes such as (*E*,*E*)-2,4-alkadienals and 4,5-epoxy-(*E*)-2-alkenals arise from the peroxidation of PUFAs [6], simpler aldehydes such as (*E*)-2-alkenals and higher molecular mass *n*-alkanals are also generated from the peroxidation of MUFAs. However, acrolein, malondialdehyde (MDA) and propanal are derived from the peroxidation of linolenoylglycerols almost exclusively [6]; these FA species were present at relatively high levels in unheated walnut oil. 

Variations in the concentrations of LOPs between the 120 min continuous and discontinuous thermo-oxidation episode time-points were also compared and are presented in Figure 9, Supplementary Materials, Figure S8. The variations were consequentially impacted by the thermo-oxidation of the FA acyl groups explained earlier in Figure 8. In the case of groundnut oil, continuous thermo-oxidation episodes for a duration of 120 min produced higher concentrations of aldehydic LOPs than those found in samples collected in its corresponding 120 min discontinuous heating protocol (Figure 9, Supplementary Materials, Figure S8). However, the 120 min discontinuous process produced higher amounts of aldehydic LOPs in ghee, extra virgin olive, macadamia, sesame, corn and walnut oils (Figure 9, Supplementary Materials, Figure S8). These observations presumably arise from the re-oxygenation of these oils during their ambient temperature ‘resting’ and cooling periods. 

According to [6], such discontinuous thermo-oxidative protocols produced higher concentrations of low- molecular-mass *n*-alkanals, 4-oxo-*n*-alkanals, *n*-alkanals and (*E*,*E*)-2,4-alkadienals than those observed for the corresponding continuous thermo-oxidation process for both olive and rapeseed oils. Nonetheless, these researchers found no significant differences in LOPs quantified in sunflower oil between these two differing protocols. Indeed, it was argued that this resistance was inherently independent of temperature since minor compounds with significant antioxidant activities may account for this effect in PUFA-rich sunflower oil [6]. However, it should also be noted that the cooling in discontinuous thermo-oxidation episodes is relatively slow, and nor was it aided by ice as previously established for the cooling of sampled thermo-oxidized oils [6].

### 4.3. Statistical Analysis of Aldehydic LOP Datasets

Full ANOVA analysis according to Model 1 revealed there were very highly significant differences between all main factors investigated (Table 4). Indeed, all main factors studied had significance values with *p* < 0.0001 for all oil FA content/IV and aldehydic LOP concentration variables tested (Table 4 and Table 5, respectively). Likewise, with the exception of only a single case, for which *p* = 0.001, the statistical significance of the first-order interaction effects was also *p* < 0.0001 for both the FA content/IV and aldehydic LOP variable datasets analyzed. 

Likewise, ANOVA performed on the corresponding Model 2 datasets again demonstrated that both the oil class and heating method main factors, and their first-order interaction effect, all exerted extremely significant effects on all aldehydic LOP variables evaluated, with *p* < 0001 in all cases. Similarly, for the FA composition/IV dataset, however, results for the ‘Between-Oil Classes’ and Heating Method × Oil Class interaction effects, all variables tested were also found to be very highly significant (*p* < 0.001), although for the ‘Between Heating Method’ factor, the oleic and linolenic content, and IV variables were not statistically significant (Table 6 and Table 7).

## 5. Conclusions

This report is part of the bulky study targeted at mapping LOPs in all oil types and classify the oils based on their susceptibilities to LOPs formation. This report compares, for the first time, the effects of continuous and discontinuous thermo-oxidation episodes on the FA contents and evolution of LOPs in ghee, groundnut, extra virgin olive, macadamia, sesame, corn and walnut oils using the high-resolution 600 MHz ^1^H NMR technique in a non-destructive fashion. In characterizing the unheated oils, ^1^H NMR data acquired clearly distinguished between the acylglycerol resonances of different classes of FAs present in the oils tested, notably their SFA, MUFA, PUFA and linolenoylglycerol contents. As expected, the degree of unsaturation of the unheated culinary oil influenced their susceptibility to thermo-oxidation. The most thermo-oxidative susceptible oils were PUFA-rich walnut, corn and sesame oils, and the least susceptible was SFA-rich ghee. In addition, thermally stressed PUFA-rich culinary oils yielded an alkenal species (signal k), which was undetectable in both MUFA- and SFA-rich culinary oils, which were exposed to these high-temperature heating periods. On completion of the thermo-oxidation duration, culinary oils thermally stressed continuously throughout a 300 min duration demonstrated higher levels of LOPs than those correspondingly heated discontinuously throughout a 480 min duration. However, in contrast, a 120 min discontinuous thermo-oxidation process resulted in higher concentrations of aldehydic LOPs than those reported for the continuous thermo-oxidation regimen. This observation may arise from the re-saturation of used oils with molecular O_2_ during these cooling periods. 

## Figures and Tables

**Table 1:** A comparison between product-label and ^1^H NMR-investigation of FA groups and IVs of culinary oils.

Culinary Oil	Product Label (% (*w*/*w*))								^1^H NMR-Derived (Molar %)							
	SFA		MUFA			PUFA	UFA	IV^u^	SFA		MUFA			PUFA	UFA	IV^u^
		ω-3	O	L	Ln	L+Ln	O+L+Ln			ω-3	O	L	Ln	L+Ln	O+L+Ln	
Ghee	67.13	-	-	-	-	0.47	-	-	72.28 ± 0.51	-	26.31 ± 0.52	1.41 ± 0.03	-	1.41 ± 0.03	27.72 ± 0.51	38.48 ± 0.12
Groundnut oil	17.14	-	59.61	-	-	18.45	78.06	-	20.99 ± 0.34	-	60.87 ± 0.64	17.81 ± 0.31	0.34 ± 0.03	18.15 ± 0.33	79.01 ± 0.34	83.23 ± 0.12
Extra virgin olive oil	16.98	-	71.30	-	-	11.72	83.02	-	15.02 ± 0.09	2.01 ± 0.05	77.66 ± 0.10	6.44 ± 0.12	0.88 ± 0.07	7.32 ± 0.18	84.98 ± 0.09	77.43 ± 0.13
Macadamia oil	12.09	-	75.82	-	-	7.95	83.77	-	16.84 ± 0.24	-	80.57 ± 0.26	2.34 ± 0.05	0.25 ± 0.02	2.58 ± 0.07	83.16 ± 0.24	75.29 ± 0.20
Sesame oil	14.60	-	-	-	-	-	-	-	17.48 ± 0.34	-	39.84 ± 0.68	42.68 ± 0.39	-	42.68 ± 0.39 *	82.52 ± 0.34	106.31 ± 0.22
Corn oil	13.46	-	29.53	-	-	57.00	86.54	-	17.91 ± 2.00	2.68 ± 0.16	31.80 ± 0.93	49.03 ± 1.09	1.26 ± 0.10	50.30 ± 1.06	82.09 ± 2.00	112.47 ± 1.37
Walnut oil	9.10	-	-	-	-	-	-	-	13.50 ± 0.30	14.25 ± 0.12	13.74 ± 0.26	59.97 ± 0.16	12.79 ± 0.05	72.76 ± 0.20	86.50 ± 0.30	145.20 ± 0.09

* Absence of Ln. ^u^IV is expressed as IV unit. Abbreviations: SFA—saturated fatty acid; MUFA—monounsaturated fatty acid; PUFA—polyunsaturated fatty acid; UFA—unsaturated fatty acid; ω-3—omega-3 fatty acid; O—oleic fatty acid; L—linoleic fatty acid; Ln—linolenic fatty acid; IV—iodine value. Values derived from ^1^H NMR data are presented as mean ± SD molar % (FA) and mean ± SD IV unit of oil.

**Table 2:** Assignments of the major intense ^1^H NMR signals of acylglycerol functions present in the ^1^H NMR profiles of unheated culinary oils, including their chemical shift values and multiplicities.

			Functional Group	
Signal	Chemical Shift (ppm)	Multiplicity	Condensed Function	Classification
A	0.780–0.839	*t*	–C**H**_3_	Saturated, oleic, and linoleic acyl groups
B’	0.856–0.893	*t*	–C**H**_3_	*n*-Butyric acid/ester acyl groups
B	0.883–0.918	*t*	–C**H**_3_	Unsaturated *ω*-3 acyl groups
C	1.119–1.344	*t*	–(C**H**_2_)*_n_*–	Bulk chain acyl groups
D	1.494–1.596	*m*	–OCO–C**H**_2_–C**H**_2_–	All acyl groups
E	1.894–2.028	*m*	–C**H**_2_–C**H**=C**H**–	All acyl groups
F	2.200–2.286	*dt*	–OCO–C**H**_2_–	All acyl groups
G	2.669–2.715	*dd*	=**H**C–C**H**_2_–C**H**=	Diunsaturated fatty acid *ω*-6 acyl groups
H	2.715–2.755	*dd*	=**H**C–C**H**_2_–C**H**=	Triunsaturated *ω*-3 acyl groups
I	4.032–4.266	*ABX (AB)*	–C**H**_2_OCOR	Glyceryl backbone functions
J	5.164–5.221	*ABX (X)*	>C**H**OCOR	Glyceryl backbone functions
K	5.221–5.347	*m*	–C**H**=C**H**–	Acyl chain olefinic functions

Abbreviations: *d*—doublet; *t*—triplet; *m*—multiplet; *dd*—doublet of doublets; *dt*—doublet of triplets; ω-3—omega-3 acyl group; ω-6—omega-6 acyl group. Letter assignments and characteristics correspond to those provided in Figure 1.

**Table 3:** Assignments of the ^1^H NMR resonances of aldehydic secondary LOPs present in the 600 MHz profiles acquired on thermally stressed culinary oils, including their chemical shift values and multiplicities.

			Functional Group	
Signal	Chemical Shift (Ppm)	Multiplicity	Condensed Function	Classification
a	9.412–9.443	*d*	–C**H**O	(*E*)-2-Alkenals
b	9.443–9.470	*d*	–C**H**O	(*E*,*E*)-2,4-Alkadienals
c	9.470–9.494	*d*	–C**H**O	4,5-Epoxy-(*E*)-alkenals
d	9.499–9.520	*d*	–C**H**O	4-Hydroxy-(*E*)-2-alkenals
e	9.507–9.523	*d*	–C**H**O	4-Hydroperoxy-(*E*)-2-alkenals
f	9.523–9.547	*d*	–C**H**O	(*Z*,*E*)-2,4-Alkadienals
g	9.661–9.697	*t*	–C**H**O	*n*-Alkanals
h	9.712–9.727	*t*	–C**H**O	4-Oxo-*n*-alkanals
i	9.733–9.748	*t*	–C**H**O	Low-molecular-mass *n*-alkanals (ethanal, propanal and *n*-butanal)
j	9.985–10.011	*d*	–C**H**O	(*Z*)-2-Alkenals
k	10.074–10.098	*d*	–C**H**O	Unidentified unsaturated aldehyde

Abbreviations: *d*—doublet*; t*—triplet. Unidentified unsaturated aldehyde is identified by ^1^H–^1^H TOCSY spectrum in Figure 4 as an alkenal species. Letter assignments and characteristics correspond to those provided in Figure 3.

**Table 4:** Statistical significance of main factors (continuous (C) versus discontinuous (D) thermal-stressing methods (C/D), oil type and heating time (min), along with their three first-order interaction effects, obtained from an ANOVA analysis of the FA composition and IV dataset (Model 1).

Factor	Oleic FA	Linoleic FA	Linolenic FA	Total ω-3 FAs	Total PUFAs	Total UFAs	Total S(+M) FAs	IV
C/D	<0.0001	<0.0001	<0.0001	<0.0001	<0.0001	<0.0001	<0.0001	<0.0001
Oil	<0.0001	<0.0001	<0.0001	<0.0001	<0.0001	<0.0001	<0.0001	<0.0001
Time (min)	<0.0001	<0.0001	<0.0001	<0.0001	<0.0001	<0.0001	<0.0001	<0.0001
C/D × Oil	<0.0001	<0.0001	<0.0001	<0.0001	<0.0001	<0.0001	<0.0001	<0.0001
C/D × Time (min)	<0.0001	<0.0001	0.001	<0.0001	<0.0001	<0.0001	<0.0001	<0.0001
Oil × Time (min)	<0.0001	<0.0001	<0.0001	<0.0001	<0.0001	<0.0001	<0.0001	<0.0001

**Table 5:** Statistical significance of main factors (continuous (C) versus discontinuous (D) thermal-stressing methods (C/D), oil type and heating time (min), along with their three first-order interaction effects, obtained from an ANOVA analysis of the aldehydic LOP dataset (Model 1).

Factor	Alkenal Species (Signal k)	(Z)-2-Alkenals	n-Alkanals (Low mwt)	4-Oxo-n-Alkanals	n-Alkanals	(Z,E)-2,4-Alkadienals	4-Hydroperoxy-(E)-2-Alkenals	4-Hydroxy-(E)-2-Alkenals	4,5-Epoxy-(E)-Alkenals	(E,E)-2,4-Alkadienals	(E)-2-Alkenals
C/D	<0.0001	<0.0001	<0.0001	<0.0001	<0.0001	<0.0001	<0.0001	<0.0001	<0.0001	<0.0001	<0.0001
Oil	<0.0001	<0.0001	<0.0001	<0.0001	<0.0001	<0.0001	<0.0001	<0.0001	<0.0001	<0.0001	<0.0001
Time (min)	<0.0001	<0.0001	<0.0001	<0.0001	<0.0001	<0.0001	<0.0001	<0.0001	<0.0001	<0.0001	<0.0001
C/D × Oil	<0.0001	<0.0001	<0.0001	<0.0001	<0.0001	<0.0001	<0.0001	<0.0001	<0.0001	<0.0001	<0.0001
C/D × Time (min)	<0.0001	<0.0001	<0.0001	<0.0001	<0.0001	<0.0001	<0.0001	<0.0001	<0.0001	<0.0001	<0.0001
Oil × Time (min)	<0.0001	<0.0001	<0.0001	<0.0001	<0.0001	<0.0001	<0.0001	<0.0001	<0.0001	<0.0001	<0.0001

**Table 6:** Statistical significance of main factors (continuous (C) versus discontinuous (D) thermal-stressing methods (C/D), oil type, and their first-order interaction effect, obtained from an ANOVA analysis of the FA composition and IV dataset (Model 2).

Factor	Oleic FA	Linoleic FA	Linolenic FA	Total ω-3 FAs	Total PUFAs	Total UFAs	Total S(+M) FAs	IV
C/D	ns	<0.0001	0.086	<0.0001	<0.0001	<0.0001	<0.0001	ns
Oil	<0.0001	<0.0001	<0.0001	<0.0001	<0.0001	<0.0001	<0.0001	<0.0001
C/D × Oil	<0.0001	<0.0001	0.032	<0.0001	<0.0001	<0.0001	<0.0001	<0.0001

Abbreviation: ns—non-significant.

**Table 7:** Statistical significance of main factors (continuous (C) versus discontinuous (D) thermal-stressing methods (C/D) and oil type, and their first-order interaction effect, obtained from an ANOVA analysis of the aldehydic LOP dataset (Model 2).

Factor	Alkenal Species (Signal k)	(Z)-2-Alkenals	n-Alkanals (Low mwt)	4-Oxo-n-alkanals	n-Alkanals	(Z,E)-2,4-Alkadienals	4-Hydroperoxy-(E)-2-Alkenals	4-Hydroxy-(E)-2-Alkenals	4,5-Epoxy-(E)-Alkenals	(E,E)-2,4-Alkadienals	(E)-2-Alkenals
C/D	<0.0001	<0.0001	<0.0001	<0.0001	<0.0001	<0.0001	<0.0001	<0.0001	<0.0001	<0.0001	<0.0001
Oil	<0.0001	<0.0001	<0.0001	<0.0001	<0.0001	<0.0001	<0.0001	<0.0001	<0.0001	<0.0001	<0.0001
C/D × Oil	<0.0001	<0.0001	<0.0001	<0.0001	<0.0001	<0.0001	<0.0001	<0.0001	<0.0001	<0.0001	<0.0001

## Data Availability

Raw data is available on request.

## Supplementary Materials

The following supporting information can be downloaded at: https://www.mdpi.com/article/10.3390/foods11131864/s1, Table S1: Assignment of the ^1^H NMR signals of minor compounds present in the ^1^H NMR profiles of unheated culinary oils (sterols, stanols and *sn*-1,2-diacylglycerol hydrolysis products), including their chemical shift values and multiplicities. Table S2: Assignment of bulk ^1^H NMR signals of CHPDs, HPMs and the olefinic resonances of α,β-UAs. Letter assignments of resonances and olefinic resonances of α,β-UAs, as well as hydroperoxide functions (primary LOPs) and formic acid present in the ^1^H NMR profiles of thermally stressed culinary oils, including their chemical shift values, and multiplicities. Table S3: Assignment of the ^1^H NMR signals of epoxy-FAs and primary alcohols present in the ^1^H NMR profiles of thermally stressed culinary oils, including their chemical shift values and multiplicities. Figure S1: ^1^H NMR spectra of minor compounds present within the 0.0–3.7 ppm regions of unheated culinary oils. Letter assignments of resonances correspond to those provided in Table 3. Figure S2: ^1^H NMR spectra showing expanded 5.5–7.0 ppm regions of (i) walnut oil thermally stressed at different times and (ii) culinary oils thermally stressed at 300 min which reveal CHPDs, HPMs, and the olefinic resonances of α,β-UAs. Letter assignments of resonances correspond to those provided in Table S2. Figure S3: ^1^H NMR spectra showing expanded 7.8–9.0 ppm regions of (i) macadamia oil and (ii) extra virgin olive oil thermally stressed for increasing times, which reveal broad resonances attributable to hydroperoxide groups (primary LOPs) and formic acid. Letter assignments of resonances correspond to those provided in Table S2. Figure S4: ^1^H NMR spectra showing expanded 2.3–4.0 ppm regions of (i) walnut oil thermally stressed at increasing time-points and (ii) culinary oils thermally stressed at 300 min, at 180 °C, which reveal resonances attributable to epoxy-FAs and primary alcohols, both types of LOP classified as secondary LOPs, although epoxy-FAs may also represent primary LOPs. Letter assignments of resonances correspond to those provided in Table S3. Figure S5: The chemical structure of 1,3,5-tribromobenzene (TBB). Figure S6: Evolution of aldehydic LOPs in thermally stressed culinary oils subjected to a continuous thermo-oxidation at 180 °C for 300 min. All values are presented as mean±SD mM (mmol LOPs per 1.0 L oil). Figure S7: Evolution of aldehydic LOPs in thermally stressed culinary oils subjected to a discontinuous thermo-oxidation at 180 °C for 480 min. All values are presented as mean ± SD mM (mmol LOPs per 1.0 L oil). Figure S8: Evolutions of aldehydic LOPs (minor types) in unheated and thermally-stressed culinary oils exposed to continuous and discontinuous thermo-oxidation episodes at 180 °C for a 120 min period. References [36,37,38,39,40,41,42,43] are cited in the supplementary materials.
