# Supplementary material for: Nuclear Magnetic Resonance Spectroscopic Analysis of the Evolution of Peroxidation Products Arising from Culinary Oils Exposed to Thermal Oxidation: An Investigation Employing 1H and 1H-1H COSY and TOCSY Techniques
Source: Foods. 2022 Jun 24;11(13):1864. doi: 10.3390/foods11131864 (PMC9265948; doi:10.3390/foods11131864)
Supplement: Supplementary file 1 [file foods-11-01864-s001.zip › foods-1754678-supplementary.pdf]

Nuclear Magnetic Resonance Spectroscopic Analysis of the Evolution of Peroxidation Products Arising from Culinary Oils Exposed to Thermal Oxidation: An Investigation Employing ^{1}H and ^{1}H-^{1}H COSY and TOCSY Techniques

Gilbert AMPEM [1], Adam LE GRESLEY [1,*], Martin GROOTVELD [2] and Declan P NAUGHTON [1]

[1] Department of Chemistry and Pharmaceutical Sciences, SEC Faculty, Kingston University, Kingston-upon-Thames, Surrey KT1 2EE, UK; g.ampem@kingston.ac.uk (G.A.); d.naughton@kingston.ac.uk (D.P.N.)
[2] Leicester School of Pharmacy, De Montfort University, Leicester LE1 9BH, UK; mgrootveld@dmu.ac.uk
* Correspondence: a.legresley@kingston.ac.uk; Tel.: +44-(0)20-8417-7432

Citation: Ampem, G.; Le Gresley, A.; Grootveld, M.; Naughton, D.P. Nuclear Magnetic Resonance Spectroscopic Analysis of the Evolution of Peroxidation Products Arising from Culinary Oils Exposed to Thermal Oxidation: An Investigation Employing ^{1}H and ^{1}H-^{1}H COSY and TOCSY Techniques. *Foods* **2022**, *11*, 1864. https://doi.org/10.3390/foods11131864

Academic Editors: Valentina Domenici, Anton Gradisek and Mecit H. Oztop

Received: 18 May 2022
Accepted: 20 June 2022
Published: 24 June 2022

Publisher's Note: MDPI stays neutral with regard to jurisdictional claims in published maps and institutional affiliations.

Copyright: © 2022 by the authors. Submitted for possible open access publication under the terms and conditions of the Creative Commons Attribution (CC BY) license (https://creativecommons.org/licenses/by/4.0/).

1. Results

1.1. Minor natural compounds detectable

Minor compounds detected in the studied culinary oils by 600 MHz ^{1}H NMR technique are profiled in Figure S1 and described in Table S1. They include sterols such as Δ7-avenasterol, β-sitosterol, Δ5-campesterol, cholesterol, Δ5-stigmasterol, and brassicasterol, as well as squalene, and *sn*-1,2-diacylglycerols. In addition, there were three unidentified signal peaks (W_1, W_2, and W_3) that varied among the studied culinary oils and these unidentified signal peaks are yet to be characterised (Figure S1). The general assignment of these signals was made according to information available in [1,2].

Signal Q, positioned at 0.603–0.618 ppm, is a singlet attributable to the methyl groups (–C**H**$_3$) of C-18 Δ7-Avenasterol (Figure S1), and was present in all studied culinary oils, although its intensity varied significantly between the oils investigated. Indeed, in decreasing order of magnitude, corn oil, sesame oil and ghee were found to contain the highest levels of this signal. Δ7-Avenasterol represents a precursor in the biosynthesis of steroids.

Signal R is a singlet ascribable to –C**H**$_3$ of C-18 β-sitosterol, Δ5-campesterol or cholesterol. Its intensity was characteristically lowest in walnut oil and highest in ghee and extra virgin olive oil (Figure S1). Although cholesterol may be present at low levels in culinary oils, higher levels of it may arise from cross-contamination from other cholesterol-rich sources such as oil extracts of animal products [3].

Signal S was detected in all analysed culinary oils and is attributable to the C18–C**H**$_3$ groups of Δ5-stigmasterol and brassicasterol (Figure S1). Δ5-Stigmasterol and brassicasterol have an antioxidant potential, and therefore, could offer a protective mechanism to oxidative damage of the unsaturated acyl groups of the culinary oils. Sterols identified in the culinary oils are 'health-friendly' and act to block the *in vivo* absorption of dietary cholesterol [4].

Signal T is a singlet (δ = 1.590–1.609 ppm) ascribable to the terminal –C**H**3 group of squalene. This resonance was observed in ghee, extra virgin olive, sesame, corn, and walnut oils (Figure S1). Squalene is a hydrocarbon and a triterpene that acts as a vital starting material for the biochemical synthesis of all sterols. Squalene also possesses viable anti-cancer and anti-ageing properties [5].

Signal U, which is ascribable to the –C**H**2OH group of *sn*-1,2-diacylglycerols, is a multiplet [1] located at 3.620–3.664 ppm in the ^{1}H NMR profiles of the oils tested (Figure S1). Amongst the culinary oils studied, ghee was found to contain the highest intensity of *sn*-1,2-diacylglycerols. Furthermore, this resonance was similar in the ^{1}H NMR spectra of sesame oil, corn oil, and walnut oils. In addition, ghee, extra virgin olive oil, and macadamia oil's *sn*-1,2-diacylglycerols signal was also similar whereas that of groundnut oil varied substantially from the other oils (Figure S1). Hydrolysis of triacylglycerols produce *sn*-1,2-diacylglycerols, along with *sn*-1.3-diacylglycerols and 1(3)- or 2-monoacylglycerols. By creating correlations between all protons within a given spin system, a ^{1}H total correlation spectroscopy (TOCSY) was previously used to show connectivity between the resonances of *sn*-1,2-diacylglycerols and *sn*-1,3-diacylglycerols in encapsulated marine oil formulations [1].

Nonetheless, [6] reported that the signal produced for *sn*-1,2- diacylglycerols by dioleoglycerols and dipalmitin on a ^{1}H NMR spectrum was a triplet whereas that characterized by mixtures of margarine lipids, dioleoglycerol, and dipalmitin was a doublet. In agreement, [7] also reported that complex mixtures of pure fatty acids and different types of fatty acids esterified to mono-, di-, and triacylglycerols produced more than one multiplet signal, which was influenced by the compositions of the mixtures. *sn*-1,2-diacylglycerols were also identified in East African virgin sunflower oils [4], along with commercially-available coconut, olive, rapeseed, and sunflower oils [8,9], and in oil extracts of commercially-available fried foods purchased in London, UK [3]. The differing forms of diacylglycerols may arise from incomplete biosynthesis of triacylglycerols, a limited catalytic activity of lipase, and/or lipolysis of triacylglycerols during extraction, processing, and/or storage of these products [1].

Table S1 Assignment of the ^{1}H NMR signals of minor compounds present in the ^{1}H NMR profiles of unheated culinary oils (sterols, stanols and *sn*-1,2-diacylglycerol hydrolysis products), including their chemical shift values and multiplicities.

			Functional group	
Signal	Chemical shift (ppm)	Multiplicity	Condensed group	Classification
Q	0.603–0.618	*s*	–C**H**$_3$ (C-18)	Δ7-Avenasterol
R	0.686–0.693	*s*	–C**H**$_3$ (C-18)	β-Sitosterol, Δ5-Campesterol, and Cholesterol
S	0.693–0.705	*s*	–C**H**$_3$ (C-18)	Δ5-Stigmasterol and Brassicasterol
T	1.590–1.609	*s*	Terminal –C**H**$_3$	Squalene
U	3.620–3.664	*d*	–C**H**$_2$OH	1,2-Diacylglycerols
W_1	0.460–0.472	*s*	Unidentified	Unidentified
W_2	0.705–0.716	*s*	Unidentified	Unidentified
W_3	0.716–0.727	*s*	Unidentified	Unidentified

Abbreviations: *s*, singlet; *m*, multiplet; *sn*, substitutional number. Letter assignments and characteristics correspond to those provided in Figure S1.

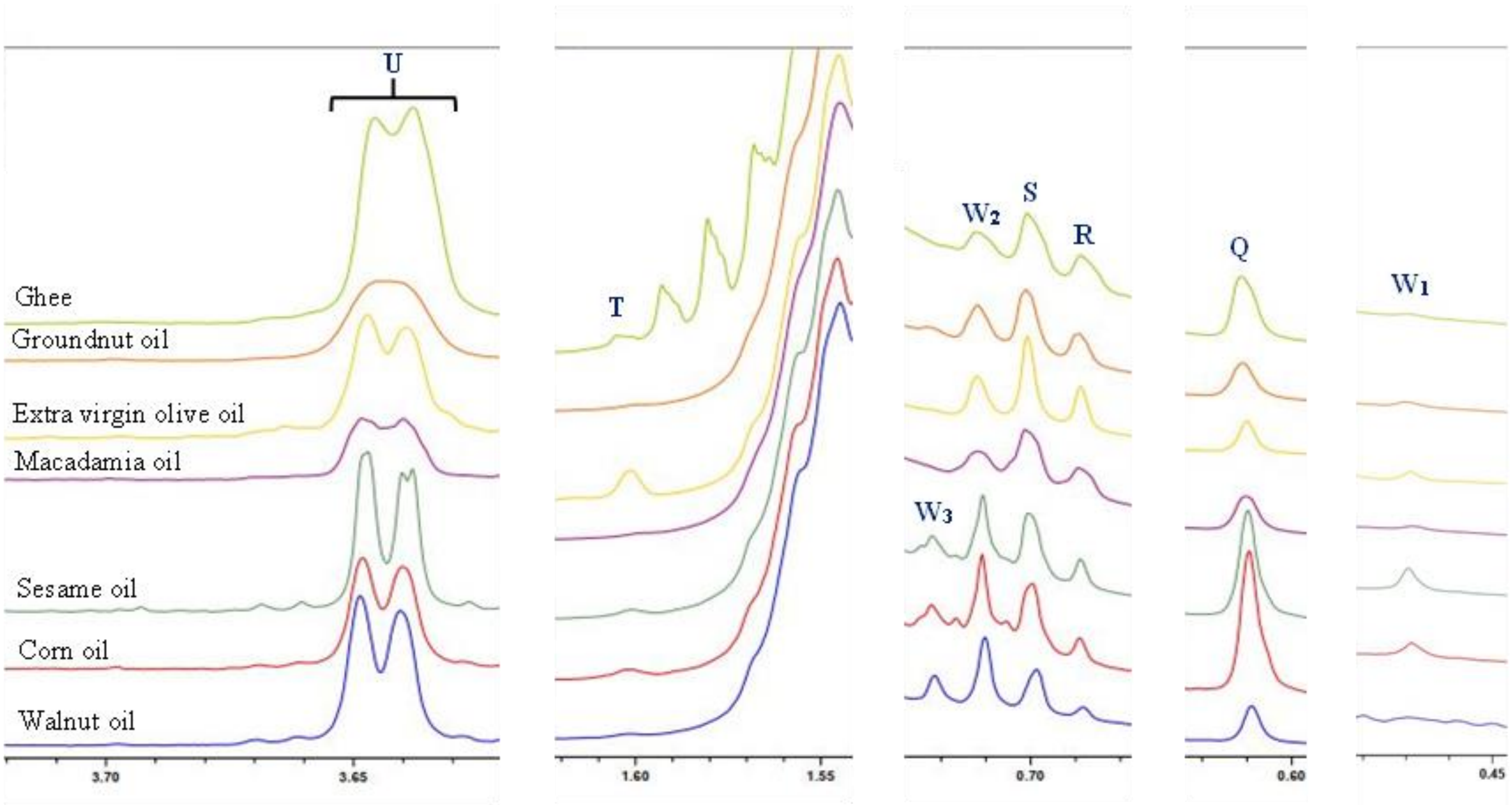

Figure S1. ^{1}H NMR spectra of minor compounds present within the 0.0–3.7 ppm regions of unheated culinary oils. Letter assignments of resonances correspond to those provided in Table 3.

1.2. Oxidation products of UFAs

1.2.1. Primary LOPs: 5.50-8.50 ppm regions of spectra acquired

1.2.1.1. Conjugated hydroperoxydienes (CHPDs) and hydroperoxymonoenes (HPMs) (primary LOPs), and the olefinic resonances of α,β-UAs (secondary LOPs)

Continuous thermo-oxidation for a period of 300 min at 180°C led to the evolution of CHPDs, HPMs, and α,β-UAs from culinary oils when exposed to high-temperature heating episodes (Figure S2ii). Resonances arising from CHPDs and HPMs, and the olefinic signals of α,β-UAs, were observed to sequentially increase with thermo-oxidation duration, as profiled in continuously thermally stressed walnut oil (Figure S2i), for example. This concurs with the previous reports of [10,11]. Amongst the oils evaluated, the highest intensity observed for signal m at 5.790–5.973 ppm and 6.710–6. 803 ppm was in sesame oil (Figure S2ii). Primary LOPs have also been identified in corn oils retained in closed receptacles and stored at room temperature for 12–121 months [12], unrefined oils extracted from pre-fermented and non-fermented cod livers [13], and in French fries purchased from two fast-food chain restaurants in the UK [3]. Subject to the oils' unsaturation degree, the intensity of the signal resonances of CHPDs, HPMs, and the olefinic resonances of α,β-UAs were, in general, in orders of PUFA-rich oils (walnut > sesame > corn oils) > MUFA-rich oils (groundnut > macadamia > extra virgin olive oils) > the only SFA-rich oil matrix (ghee) (Figure S2ii).

The differences in the ^{1}H NMR detectable CHPDs, HPMs, and olefinic resonances of α,β-UAs were critically dependent on variations in the degree of unsaturation of the oils. Amongst the oil samples tested, the signal resonances of CHPDs, HPMs, and olefinic resonances of α,β-UAs were, as expected, found to be in order of PUFA-rich sunflower > MUFA-rich olive and rapeseed > SFA-rich coconut oils [11]. Primary LOPs were largely ^{1}H NMR-undetectable in coconut oil, which contains ca. 95 molar % SFAs [11]. At high-temperature frying episodes, primary LOPs are chemically

unstable, short-lived intermediates that fragment to a wide and diverse range of secondary LOPs, which includes alcohols, oxoacids, aldehydes, and ketones [14].

(i)

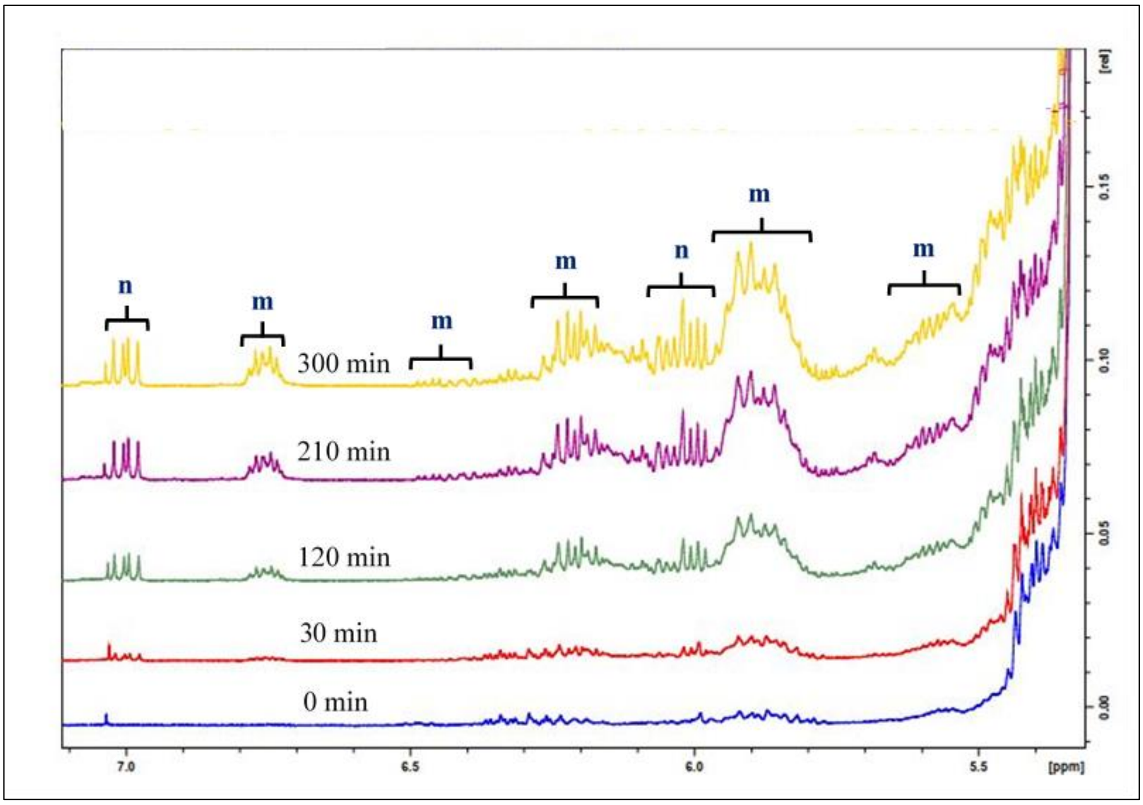

(ii)

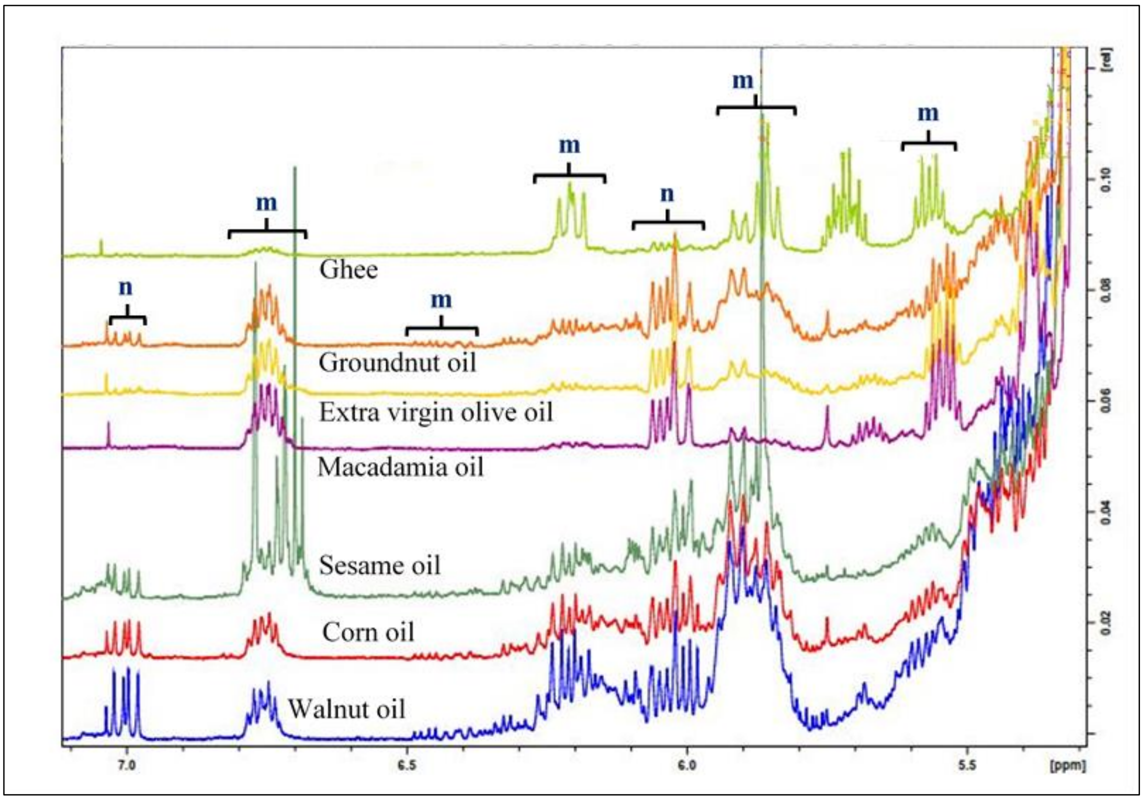

Figure S2. ^{1}H NMR spectra showing expanded 5.5–7.0 ppm regions of (i) walnut oil thermally stressed at different times and (ii) culinary oils thermally stressed at 300 min which reveal CHPDs, HPMs, and the olefinic resonances of α,β-UAs. Letter assignments of resonances correspond to those provided in Table S2.

Table S2 Assignment of bulk ^{1}H NMR signals of CHPDs, HPMs and the olefinic resonances of α,β-UAs. Letter assignments of resonances and olefinic resonances of α,β-UAs, as well as hydroperoxide functions (primary LOPs) and formic acid present in the ^{1}H NMR profiles of thermally stressed culinary oils, including their chemical shift values, and multiplicities.

		Functional group		
Signal	Chemical shift (ppm)	Multiplicity	Condensed function	Classification
m	5.505–5.594	*ddm*	–C**H**═C**H**–C**H**═C**H**–	(*Z*,*E*)-conjugated olefinic protons of (*Z*,*E*)-2,4-alkadienals and (*Z*)-2-alkenals
m	5.790–5.973	*ddm*	–C**H**═C**H**–C**H**═C**H**–	
m	6.158–6.277	*ddd*	–C**H**═C**H**–C**H**═C**H**–	(*E*,*E*)-conjugated olefinic protons of 4,5-epoxy-(*E*)-alkenals and (*Z*)-2-alkenals
m	6.365–6.493	*ddtd*	–C**H**═C**H**–C**H**═C**H**–	
m	6. 710–6.803	*ddm*	–C**H**═C**H**–C**H**═C**H**–	(*E*,*E*)-conjugated olefinic protons of (*E*)-2-alkenals, 4-hydroxy-(*E*)-2-alkenals, 4-hydroperoxy-(*E*)-2-alkenals, and UUA (signal k)
n	5.972–6.072	*ddm*	–C**H**═C**H**–C**H**═C**H**–	(*E*,*E*)-conjugated olefinic protons of (*E*)-2-alkenals
n	6.966–7.031	*dddd*	–C**H**═C**H**–C**H**═C**H**–	(*E*,*E*)-conjugated olefinic protons of (*E*,*E*)-2,4-alkadienals
p	8.000–8.500	-	–OO**H**	Hydroperoxide functions
x	7.935–7.942	*s*	–CO**H**	Formic acid

Abbreviations: *s*, singlet; *d*, doublet; *t*, triplet; *m*, multiplet; *dd*, double doublets; Letter assignments and characteristics correspond to those provided in Figures S2 and S3.

1.2.1.2. Hydroperoxides and formic acid

Hydroperoxide groups containing conjugated diene functions are important precursors to the formation of *n*-alkanals, (*E*)-2-alkenals, and (*E*,*E*)-2,4-alkadienals, and 4-hydroxy-(*E*)-2-alkenals, amongst other aldehydes [15]. In the culinary oils subjected to a 300 min continuous thermo-oxidation duration, the ^{1}H NMR resonances of hydroperoxide (–OO**H**) protons, which were located within the 8.0–8.5 ppm chemical shift range in spectra, were detectable in ghee, and extra virgin olive and macadamia oils at high levels, but undetectable in the PUFA-rich culinary oils (sesame, corn, and walnut oils) (Figure S3iii). However, significant of –OO**H** resonances were also identified in groundnut oil at the 300 min thermo-oxidation time-point (Figure S3iii). These resonances were ^{1}H NMR undetectable at the 30 min time-point in macadamia oil, and this indicates that those detectable prior to the heating process decomposed to secondary LOPs, including aldehydes, but then were further generated from time-points lying between 30 and 120 min (Figure S3i). Notwithstanding, these resonances evolved with increasing thermo-oxidation duration at a constant temperature of 180°C (Figure S3i). However, a converse effect was observed in thermally stressed extra virgin olive oil. Intriguingly, no fewer than seven or so of these –OO**H** function resonances were detectable in this MUFA-rich oil, and this observation was an indication that high-resolution ^{1}H NMR analysis has the ability to distinguish between ranges of structurally different hydroperoxides in this oil. Indeed, the differential MUFA and PUFA sources of these hydroperoxides in this product may account for this. The signal resonances of these hydroperoxide functions decreased over the 300 min of thermally stressing extra virgin-olive oil at 180°C (Figure S3ii). However, their signal intensities were similar across the sampling times in both unheated ghee and the thermo-oxidation of ghee at 180°C.

The ^{1}H NMR detection of lipid hydroperoxide functions have been previously reported in thermo-oxidized coconut, olive, rapeseed, and sunflower oils at 180°C [11,15,16]. They were also detected in 'neat' culinary oil carefully extracted from French fries and purchased from a fast-food restaurant [3]. At a constant temperature of 180°C, the resonances of these hydroperoxide functions increased as a function of thermo-oxidation duration in coconut oil [11]. A similar observation was

made for olive oil, however, at the 210 min thermo-oxidation duration time-points, a gradual reduction in these resonance intensities in olive oil was observed [11]. In thermally stressed rapeseed and sunflower oils, these hydroperoxide resonances were not visible in the unheated products. However, the intensity of these signals was prominent at 210 and 30 min sampling time-points of continuously thermo-oxidised rapeseed and sunflower oils, respectively [11]. Furthermore, the resonances of hydroperoxide functions decreased in sunflower oil as thermo-oxidation duration increased [11]. Hydroperoxide functions have also been reported in sunflower oil heated at 70°C and 100°C [10], and also in corn oil retained in tightly sealed receptacles stored at room temperature for a period of 12–121 months [12].

The production of signal x (formic acid) in the thermally stressed oils arises from the decomposition of malondialdehyde (MDA), which is derived from the peroxidation of ω-3 acyl groups. Signal x was observed to evolve with prolonged thermo-oxidation episodes, and this is demonstrated in macadamia oil (Figures S3i) and extra virgin olive oil (Figures S3ii). Indeed, this tertiary LOP was identified in all thermo-oxidised culinary oils, except for SFA-rich ghee (Figure S3iii). The intensity of the resonance of signal x varied amongst the experimented thermo-oxidised culinary oils, with extra virgin olive, corn, and walnut oils showing the lowest intensity (Figure S3iii). Notwithstanding, these intensities were relatively higher in thermo-oxidised groundnut macadamia, and sesame oils (Figure S3iii). This was, in spite of, the fact that ω-3 acyl groups were undetected in the culinary oils that had higher intensity of signal x. By implication, it is reasonable to conclude that some aldehydic LOPs, other than MDA may be acting as precursors for the evolution of signal x in the oils.

(i)

(ii)

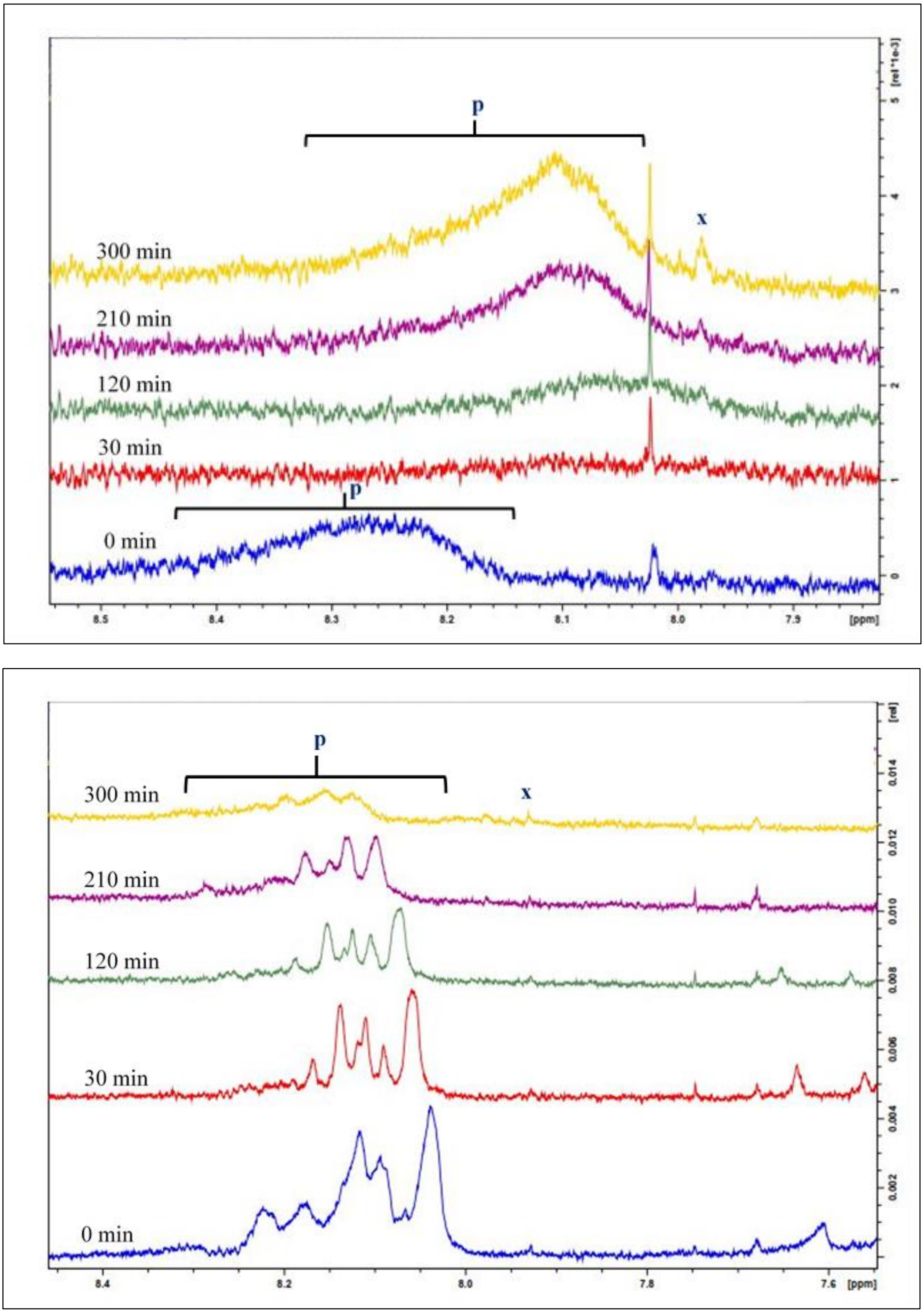

Figure S3. ^{1}H NMR spectra showing expanded 7.8–9.0 ppm regions of (i) macadamia oil and (ii) extra virgin olive oil thermally stressed for increasing times, which reveal broad resonances attributable to hydroperoxide groups (primary LOPs) and formic acid. Letter assignments of resonances correspond to those provided in Table S2.

(iii)

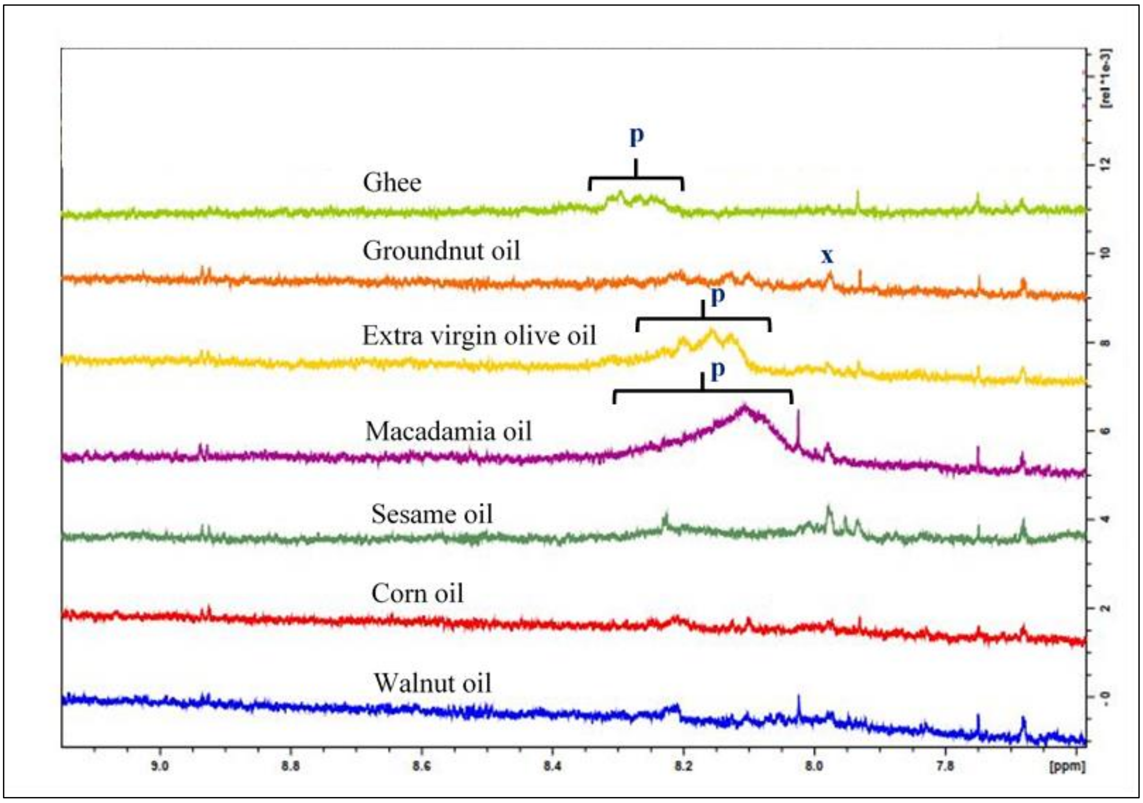

Figure S3. (Continued) ^{1}H NMR spectra showing expanded 7.8–9.0 ppm regions of (iii) culinary oils thermally stressed at 300 min which reveal broad resonances attributable to hydroperoxide groups (primary LOPs) and formic acid. Letter assignments of resonances correspond to those provided in Table S2.

1.2.2. Secondary LOPs

1.2.2.1. Epoxy-FAs and primary alcohols

The thermo-oxidation of oleic and linoleic FAs have been reported to generate epoxy-FAs [2,10]. In the current study, at a constant temperature of 180℃, the intensity of the signal resonances of epoxy-FAs (signals q, r, s, t, u, and z) and primary alcohols (signal v) increased as a function of thermo-oxidation duration for all culinary oils investigated (Figure S4i). Signal v were, however, ^{1}H NMR detectable majorly in groundnut, corn, and walnut oils, with its highest and lowest intensities characterised in walnut and groundnut oils, respectively (Figure S4ii). Traces of signal v were also observed in ghee and sesame oil (Figure S4ii). The evolution of epoxy-FAs in thermally stressed culinary oils were dependent on the oil's unsaturation degree. As expected, SFA-rich ghee produced

relatively lower intensities of epoxy-FAs than those found in MUFA-rich culinary oils (groundnut, extra virgin olive, and macadamia oils), the intensities of which were relatively lower than those observed in PUFA-rich oils (sesame, corn, and walnut oils) (Figure S4ii).

Amongst the epoxy-FAs identified, (*E*)-9,10-epoxystearate (signal q) identified by its –C**HO****H**C– protons, was ^{1}H NMR detectable in trace amounts in PUFA-rich sesame, corn, and walnut oils, as well as in SFA-rich ghee (Figure S4ii). Signal t, characterized by the –C**HO****H**C– protons (δ = 2.927–3.092 ppm) in the ^{1}H NMR profiles acquired, is ascribable to superimposed 9,10-Epoxy-octadecanoate, 9,10-Epoxy-12-octadecenoate (leukotoxin), and 12,13-Epoxy-9-octadecenoate (isoleukotoxin) signals (Figure S4ii). Leukotoxin and isoleukotoxin derivatives have been suggested to cause malfunction of healthy organs and breast cancer cell proliferation, as well as the degeneration of leukocytes, a process ultimately leading to cell death [17,18]. These were also reported in oil extracts of commercially-available French fry samples purchased in the UK [3]. Epoxy-FA identified in this study have also been reported in thermally stressed sunflower [10] and extra virgin olive oils [19], as well as in continuous, discontinuous, and partially-substituted thermo-oxidised coconut , olive, rapeseed, and sunflower oils [11]. In the report by [8], increasing concentrations of polydimethylsiloxane (PDMS) were demonstrated to suppress the evolution of epoxy-FAs and primary alcohols in a concentration- dependent manner, in both unstirred and 250 rpm-stirred sunflower oils thermally stressed continuously at 180°C.

(i)

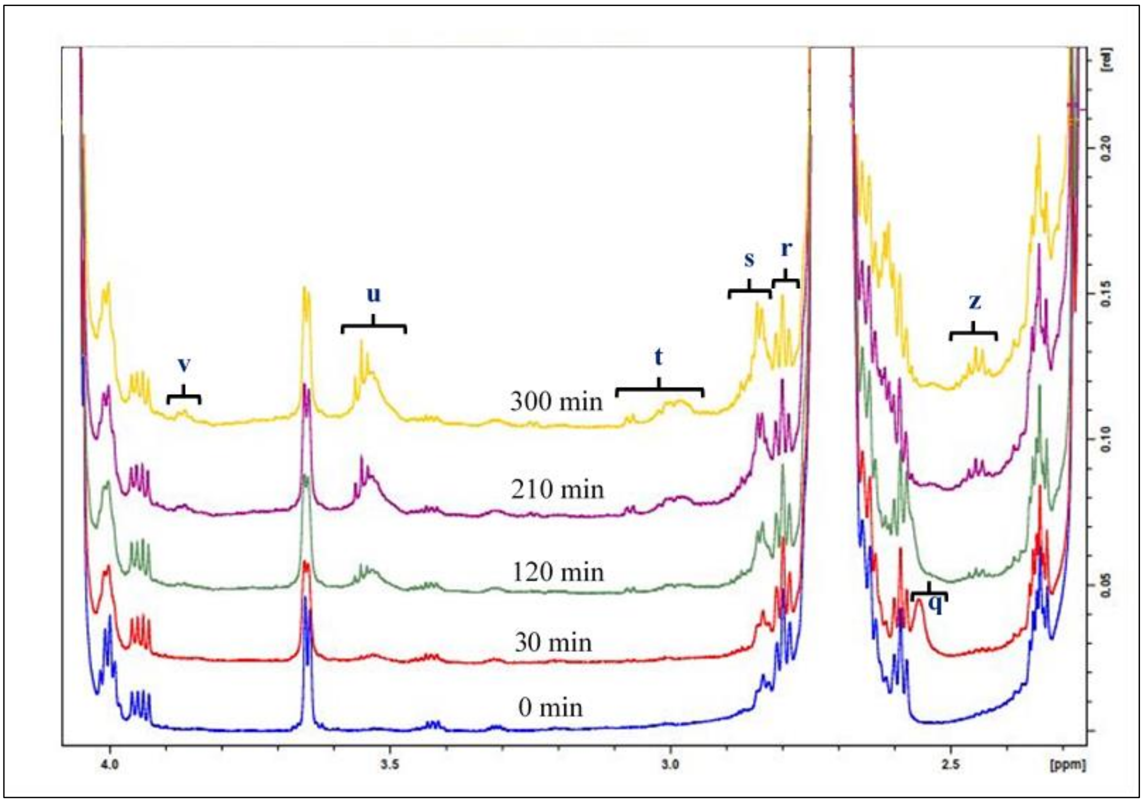

(ii)

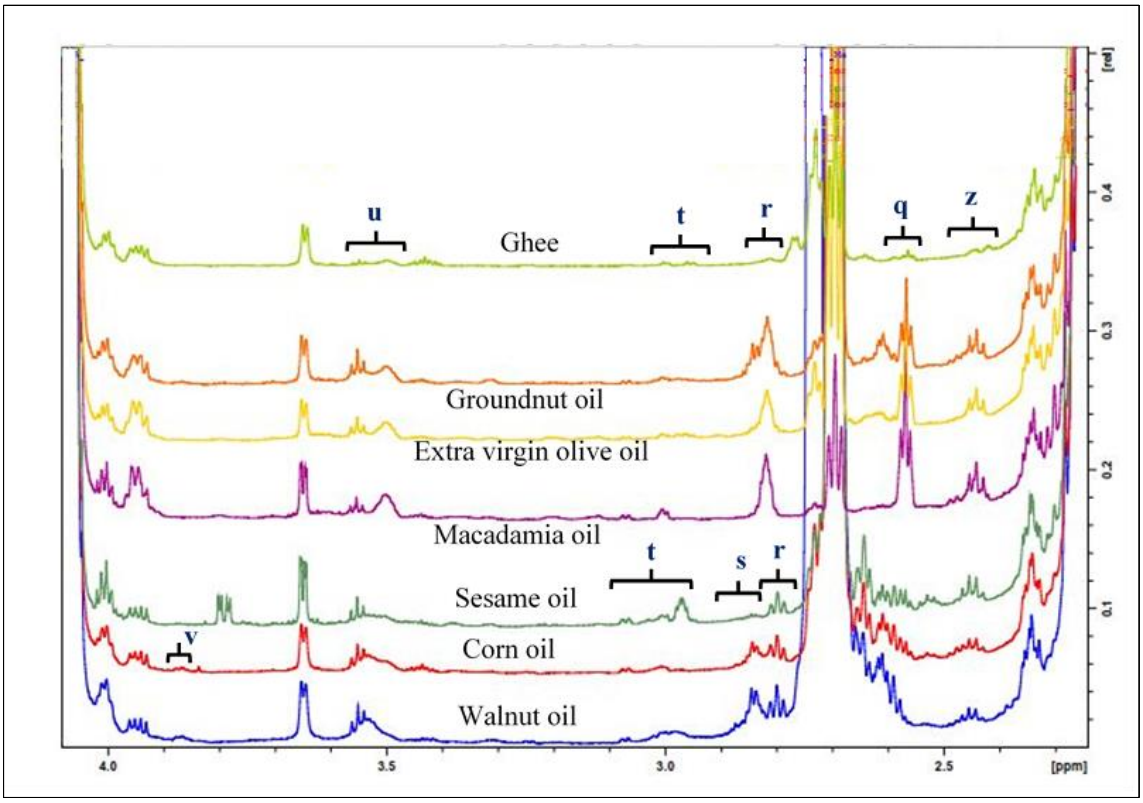

Figure S4. ^{1}H NMR spectra showing expanded 2.3–4.0 ppm regions of (i) walnut oil thermally stressed at increasing time-points and (ii) culinary oils thermally stressed at 300 min, at 180°C, which reveal resonances attributable to epoxy-FAs and primary alcohols, both types of LOP classified as secondary LOPs, although epoxy-FAs may also represent primary LOPs. Letter assignments of resonances correspond to those provided in Table S3.

Table S3 Assignment of the ^{1}H NMR signals of epoxy-FAs and primary alcohols present in the ^{1}H NMR profiles of thermally stressed culinary oils, including their chemical shift values and multiplicities.

			Functional group	
Signal	Chemical shift (ppm)	Multiplicity	Condensed function	Classification
z	2.395–2.484	*m*	-	Unidentified
q	2.537–2.589	*m*	–C**H**O**H**C–	(*E*)-9,10-Epoxystearate
r	2.780–2.820	*m*	–C**H**O**H**C–	(*Z*)-9,10-Epoxystearate
s	2.820–2.878	*m*	–C**H**OHC–CHO**H**C–	9,10–12,13-Diepoxyoctadecanoate
t	2.927–3.092	*m*	–C**H**O**H**C–	9,10-Epoxy-octadecanoate; 9,10-Epoxy-12-octadecenoate (leukotoxin); and 12,13-Epoxy-9-octadecenoate (isoleukotoxin)
u	3.465–3.585	*m*	–CHO**H**C–CH_2–C**H**OHC–	9,10–12,13-Diepoxyoctadecanoate
v	3.846–3.888	*m*	α-C$\mathbf{H}_2$	Primary alcohol LOPs

Abbreviations: *m*, multiplet. Letter assignments and characteristics correspond to those provided in Figure S4.

2. Discussion

2.1. Aldehydic LOPs

The concentrations of ^{1}H NMR detectable aldehydic LOPs in culinary oils subjected to a continuous and discontinuous thermo-oxidation processes were derived with respect to an internal standard (1,3,5-tribromobenzene (TBB)). On the ^{1}H NMR, the resonance of TBB at 7.600–7.615 ppm was integrated and calibrated as 3, which is its total number of protons (Figure S5). This was then used as the baseline for the integration of all aldehydic resonances. The concentrations of aldehydes were then derived by multiplying the integral values of the aldehydes by 1.1 mM, which is the concentration of TBB in the 0.6 mL TBB-$C^{2}HCl_3$-oil mixture in the NMR tube.

As a result, the derived concentrations of aldehydes were presented in millimolar (mM), which also is mmol LOPs per 1.0 L oil. Figures S6, S7 are graphical presentations of aldehydic LOPs quantified in continuous and discontinuous thermo-oxidised culinary oils, respectively. Comparisons of 120 min time-points for 4-oxo-*n*-alkanals, *n*-alkanals (low mwt), (*Z*)-2-alkenals, and alkenal species (signal k) are also shown in Figure S8. These concentrations of aldehydic LOPs are discussed in the Manuscript under section 4.2.

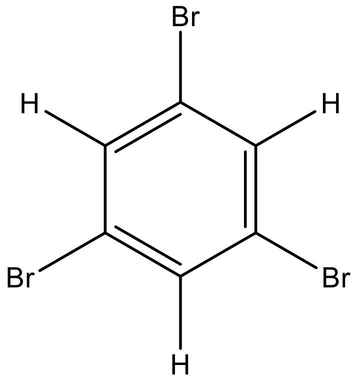

Figure S5. The chemical structure of 1,3,5-tribromobenzene (TBB).

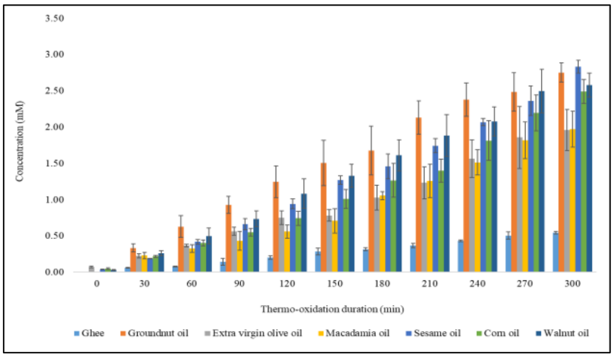

(*E*)-2-Alkenals

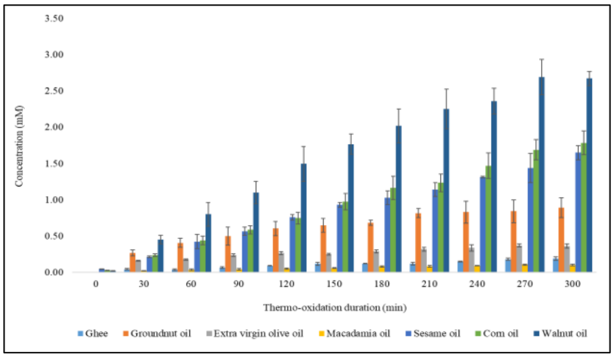

(*E*,*E*)-2,4-Alkadienals

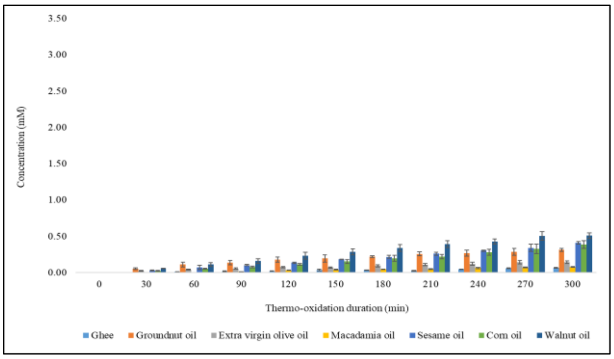

4,5-Epoxy-(*E*)-alkenals

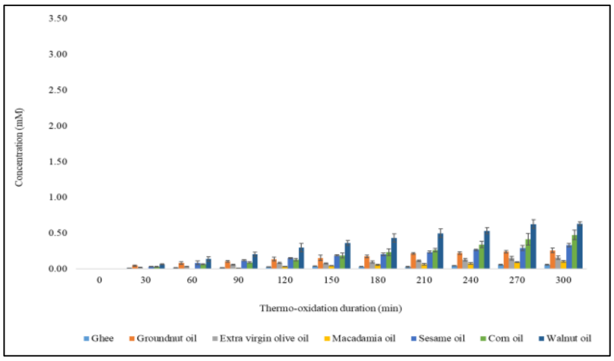

4-Hydroxy-(*E*)-2-alkenals

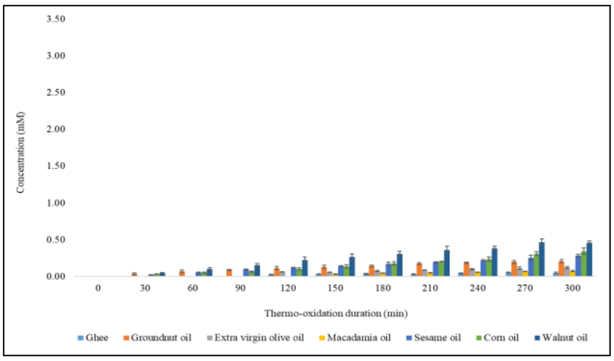

4-Hydroperoxy-(*E*)-2-alkenals

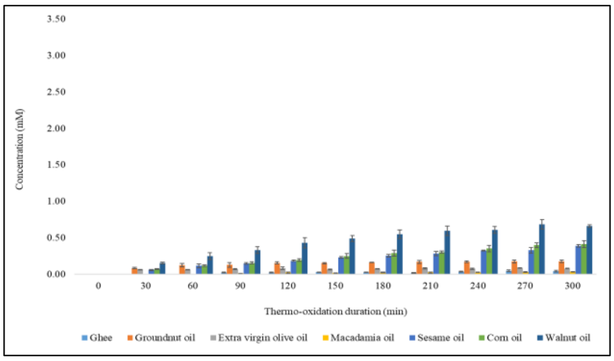

(*Z*,*E*)-2,4-Alkadienals

Figure S6. Evolution of aldehydic LOPs in thermally stressed culinary oils subjected to a continuous thermo-oxidation at 180°C for 300 min. All values are presented as mean±SD mM (mmol LOPs per 1.0 L oil).

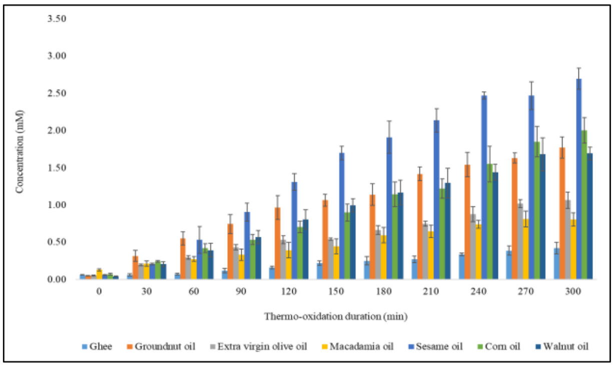

n-Alkanals

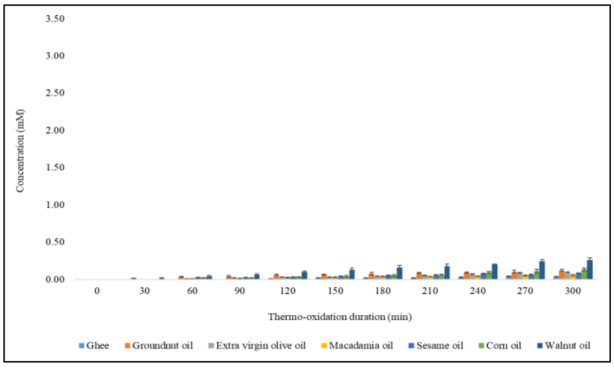

4-Oxo-*n*-alkanals

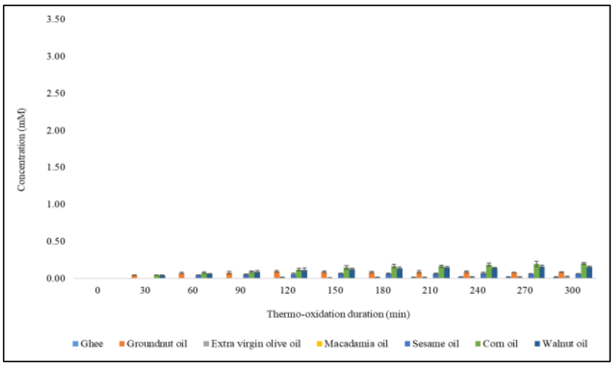

n-Alkanals of low molecular weight

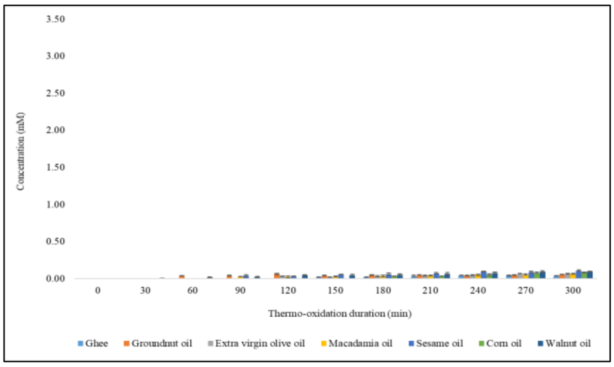

(*Z*)-2-Alkenals

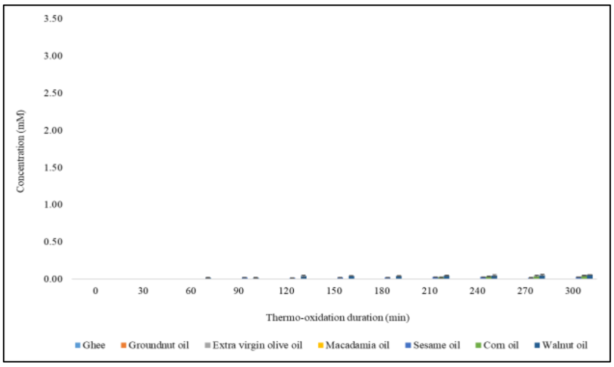

Alkenal species (signal k)

Figure S6. (*Continued*) Evolution of aldehydic LOPs in thermally stressed culinary oils subjected to a continuous thermo-oxidation at 180°C for 300 min. All values are presented as mean±SD mM (mmol LOPs per 1.0 L oil).

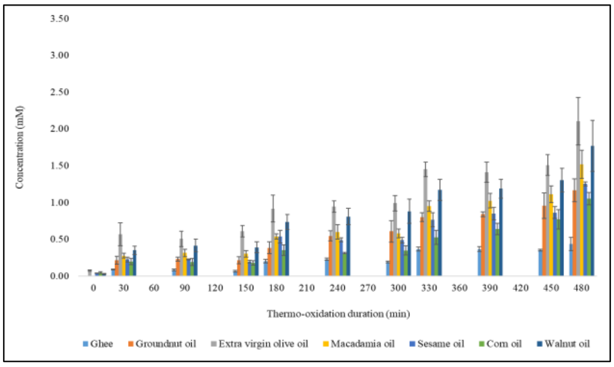

(*E*)-2-Alkenals

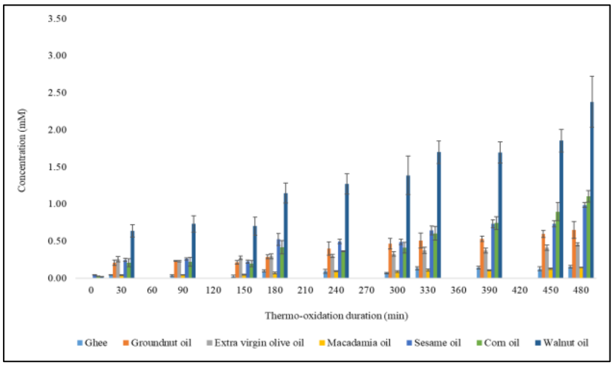

(*E*,*E*)- 2,4-Alkadienals

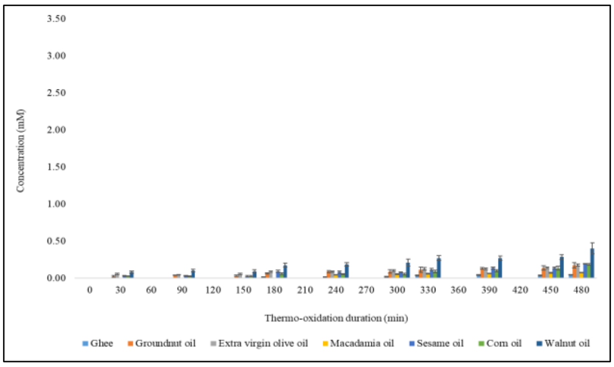

4,5-Epoxy-(*E*)-alkenals

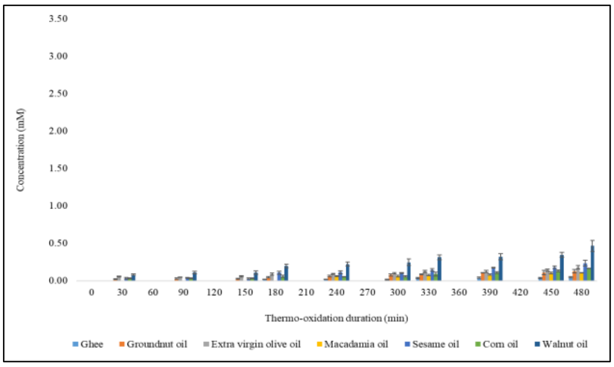

4-Hydroxy-(*E*)-2-alkenals

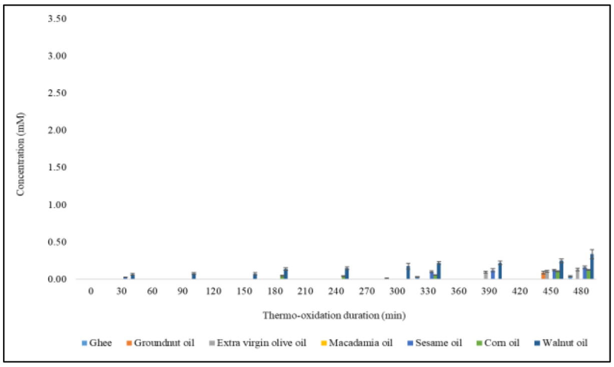

4-Hydroperoxy-(*E*)-2-alkenals

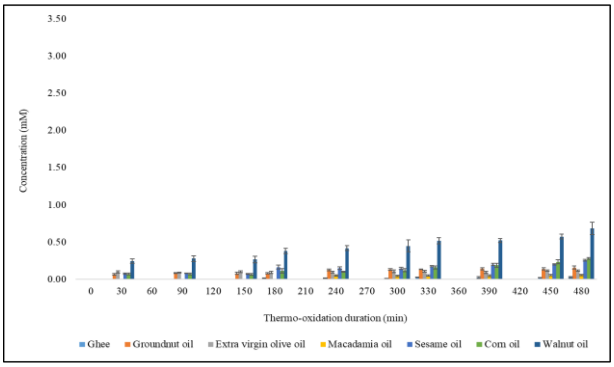

(*Z*,*E*)-2,4-Alkadienals

Figure S7. Evolution of aldehydic LOPs in thermally stressed culinary oils subjected to a discontinuous thermo-oxidation at 180°C for 480 min. All values are presented as mean±SD mM (mmol LOPs per 1.0 L oil).

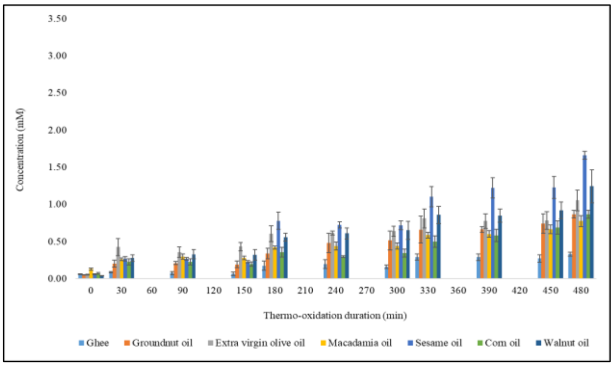

n-Alkanals

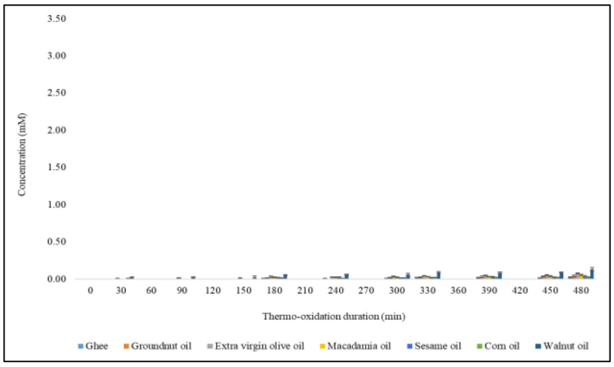

4-Oxo-*n*-alkanals

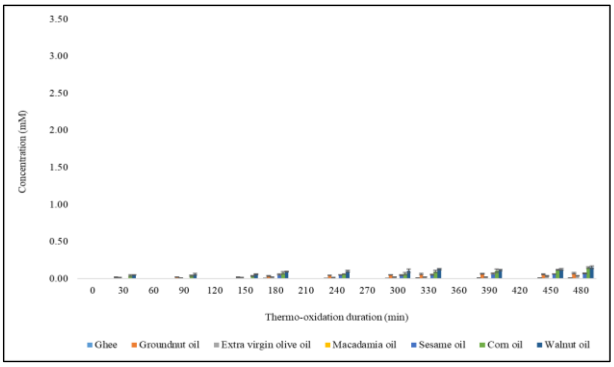

n-Alkanals of low molecular weight

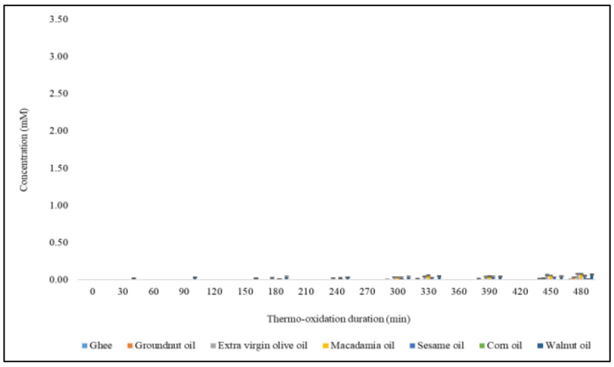

(*Z*)-2-Alkenals

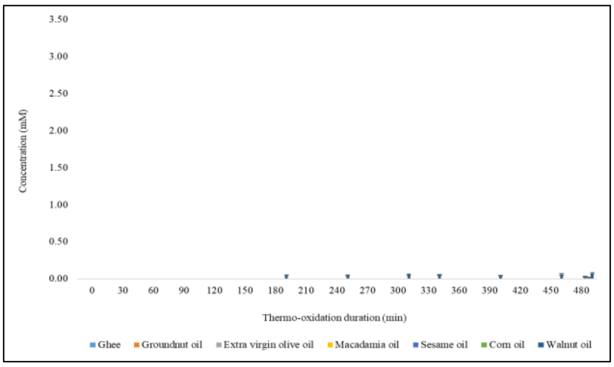

Alkenal species (signal k)

Figure S7. (*Continued*) Evolution of aldehydic LOPs in thermally stressed culinary oils subjected to a discontinuous thermo-oxidation at 180°C for 480 min. All values are presented as mean±SD mM (mmol LOPs per 1.0 L oil)

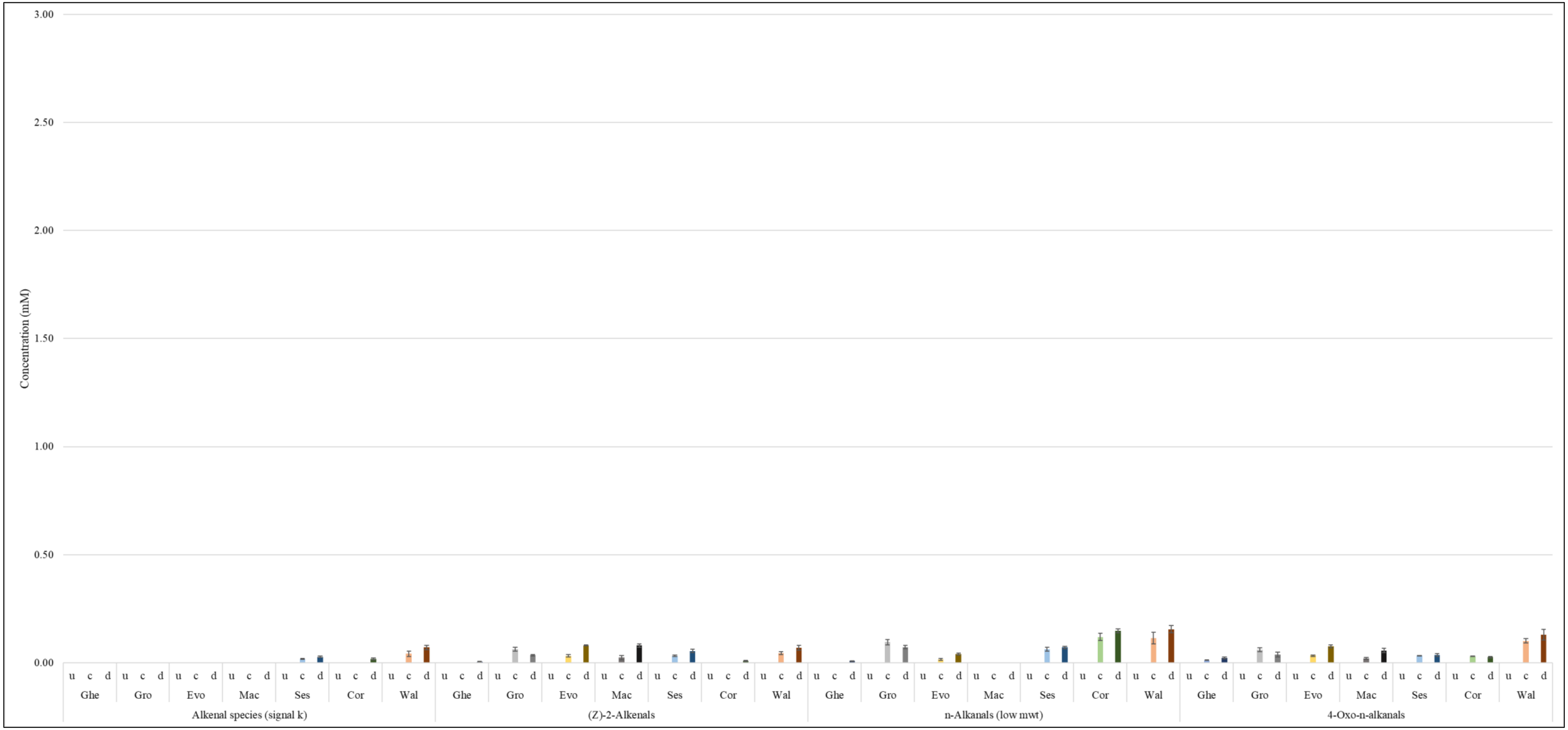

Figure S8. Evolutions of aldehydic LOPs (minor types) in unheated and thermally-stressed culinary oils exposed to continuous and discontinuous thermo-oxidation episodes at 180°C for a 120 min period. Abbreviations: u, unheated oil; c, continuous thermo-oxidation episode; d, discontinuous thermo-oxidation episode; Ghe, ghee; Gro, groundnut oil; Evo, extra virgin olive oil; Mac, macadamia oil; Ses, sesame oil; Cor, corn oil; Wal, walnut oil. The values are presented as mean±SD mM (mmol LOPs per 1.0 L volume of oil).

References

1. Siddiqui, N.; Sim, J.; Silwood, C.J.L.; Toms, H.; Iles, R.A. Grootveld, M. Multicomponent analysis of encapsulated marine oil supplements using high-resolution ^{1}H and ^{13}C NMR techniques. *J. Lipid Res*. **2003**, 44, 2406–2427. doi.org/10.1194/jlr.D300017-JLR200.
2. Martínez-Yusta, A.; Goicoechea, E.; Guillén, M.D. A review of thermo-oxidative degradation of food lipids studied by ^{1}H NMR Spectroscopy: Influence of degradative conditions and food lipid nature. *Compr. Rev. Food Sci. Food Saf.* **2014**, 13, 838–859. doi.org/10.1111/1541-4337.12090.
3. Le Gresley, A.; Ampem, G.; De Mars, S.; Grootveld, M.; Naughton, D.P. "Real-world" evaluation of lipid oxidation products and trace metals in French fries from two chain fast-food restaurants. *Front. nutr*. **2021**, 8, 620952. doi: 10.3389/fnut.2021.620952.
4. Percival B.; Savel E.; Ampem G.; Gibson M.; Edgar, M.; Jafari F.; Woodason K.; Frederick K.; Wilson P. Grootveld M. Molecular composition of and potential health benefits offered by natural East African virgin sunflower oil products: A 400 MHz ^{1}H NMR analysis study. *Int. J. Nutr*. **2019**, 3, 22–43. doi: 10.14302/issn.2379-7835.ijn-19-2677.
5. De Stefani, E.; Ronco, A. Squalene: A multi-task link in the crossroads of cancer and aging. *Funct. food health dis.* **2013**, 3, 462–476. doi: 10.31989/ffhd.v3i12.30.
6. Sopelana, P.; Arizabaleta, I.; Ibargoitia, M.L.; Guillén, M.D. Characterization of the lipidic components of margarines by ^{1}H nuclear magnetic resonance. *Food Chem*. **2013**, 141, 3357–3364. doi: 10.1016/j.foodchem.2013.06.026.
7. Nieva-Echevarría, B.; Goicoechea, E.; Manzanos, M.J.; Guillén, M.D. A method based on ^{1}H NMR spectral data useful to evaluate the hydrolysis level in complex lipid mixtures. *Food Res. Int.* **2014**, 66, 379–387. doi.org/10.1016/j.foodres.2014.09.031.
8. Ampem, G.; Le Gresley, A.; Grootveld, M.; Naughton, D.P. The role of polydimethylsiloxane in suppressing the evolution of lipid oxidation products in thermo-oxidised sunflower oil: Influence of Stirring Processes. *Front. nutr*. **2021**, doi: 10.3389/fnut.2021.721736.

9. Ampem, G.; Le Gresley, A.; Grootveld, M.; De Mars, S.; Naughton, D.P. The impact of partial oil substitution and trace metal ions on the evolution of peroxidation products in thermally stressed culinary oils. *Food Chem*. **2022**, 375, doi.org/10.1016/j.foodchem.2021.131823.
10. Goicoechea, E.; Guillén, M.D. Analysis of hydroperoxides, aldehydes and epoxides by ^{1}H Nuclear Magnetic Resonance in sunflower oil oxidized at 70 and 100°C. *J. Agric. Food Chem.* **2010**, 58, 6234–6245. doi: 10.1021/jf1005337.
11. Le Gresley, A.; Ampem, G.; Grootveld, M.; Percival, B.C.; Naughton, D.P. Characterisation of peroxidation products arising from culinary oils exposed to continuous and discontinuous thermal degradation processes. *Food Funct*. **2019**, 10, 7952–7966. doi: 10.1039/C9FO02065A.
12. Guillén, M.D.; Goicoechea, E. Oxidation of corn oil at room temperature: Primary and secondary oxidation products and determination of their concentration in the oil liquid matrix from ^{1}H nuclear magnetic resonance data. *Food Chem*. **2009**, 116, 183–192. doi.org/10.1016/j.foodchem.2009.02.029.
13. Percival, B.C.; Wann, A.; Zbasnik, R.; Schlegel, V.; Edgar, M.; Zhang, J.; Ampem, G.; Wilson, P.; Le Gresley, A.; Naughton, D.; Grootveld, M. Evaluations of the peroxidative susceptibilities of cod liver oils by a ^{1}H NMR analysis strategy: Peroxidative resistivity of a natural collagenous and biogenic amine-rich fermented product. *Nutrients* **2020**, 12, 753. doi: 10.3390/nu12030753.
14. Wann, A.I.; Percival, B.C.; Woodason, K.; Gibson, M.; Vincent, S.; Grootveld, M. Comparative ^{1}H NMR-based chemometric evaluations of the time-dependent generation of aldehydic lipid oxidation products in culinary oils exposed to laboratory-simulated shallow frying episodes: Differential patterns observed for omega-3 fatty acid-containing soybean oils. *Foods* **2021**, 10, doi.org/10.3390/foods10102481.
15. Claxson, A.W.D.; Hawkes, G.E.; Richardson, D.P.; Naughton, D.P.; Haywood, R.M.; Chander, C. L.; Atherton, M.; Lynch, E.J.; Grootveld, M.C. Generation of lipid peroxidation

products in culinary oils and fats during episodes of thermal stressing: a high field ^{1}H NMR study. *FEBS Lett*. **1994**, 355, 81–90. doi: 10.1016/0014-5793(94)01147-8.

16. Haywood, R.M.; Claxson, A.W.D.; Hawkes, G.E.; Richardson, D.P.; Naughton, D.P.; Coumbarides, G.; Hawkes, J.; Lynch, E.J.; Grootveld, M.C. Detection of aldehydes and their conjugated hydroperoxydiene precursors in thermally-stressed culinary oils and fats: investigations using high resolution proton NMR spectroscopy. *Free Radic. Res.* **1995**, 22, 441–482. doi: 10.3109/10715769509147552.
17. Markaverich, B.M.; Crowley, J.R.; Alejandro, M.A.; Shoulars, K.; Casajuna, N.; Mani, S.; Reyna A.; Sharp, J. Leukotoxins diols from ground corncob bedding disrupt estrus cyclicity in rats and stimulate MCF-7 breast cancer cell proliferation. *Environ. Health Perspect*. **2005**, 113, 1698–1704. doi: 10.1289/ehp.8231.
18. Thompson, D.A.; Hammock, B.D. Dihydroxyoctadecamonoenoate esters inhibit the neutrophil respiratory burst. *J. Biosci*. **2007**, 32, 279–291. doi: 10.1007/s12038-007-0028-x.
19. Guillén, M.D.; Uriarte, P.S. Study by ^{1}H NMR spectroscopy of the evolution of extra virgin olive oil composition submitted to frying temperature in an industrial fryer for a prolonged period of time. *Food Chem*. **2012**, 134, 162–172. doi.org/10.1016/j.foodchem.2012.02.083.
